# Comparison of a preoperative MR-based recurrence risk score versus the postoperative score and four clinical staging systems in hepatocellular carcinoma: a retrospective cohort study

**DOI:** 10.1007/s00330-022-08811-6

**Published:** 2022-05-13

**Authors:** Hong Wei, Hanyu Jiang, Yun Qin, Yuanan Wu, Jeong Min Lee, Fang Yuan, Tianying Zheng, Ting Duan, Zhen Zhang, Yali Qu, Jie Chen, Yuntian Chen, Zheng Ye, Shan Yao, Lin Zhang, Ting Yang, Bin Song

**Affiliations:** 1 Department of Radiology, West China Hospital, Sichuan University, No. 37, GUOXUE Alley, Chengdu, 610041 Sichuan China; 2 Big Data Research Center, University of Electronic Science and Technology of China, Chengdu, Sichuan China; 3 Department of Radiology, Seoul National University College of Medicine, Seoul, South Korea; 4 Department of Radiology, Sanya People’s Hospital, Sanya, Hainan China

**Keywords:** Carcinoma, hepatocellular, Recurrence, Magnetic resonance imaging, Gadolinium ethoxybenzyl DTPA, Hepatectomy

## Abstract

**Objectives:**

To establish a risk score integrating preoperative gadoxetic acid–enhanced magnetic resonance imaging (EOB-MRI) and clinical parameters to predict recurrence after hepatectomy for patients with hepatocellular carcinoma (HCC) and to compare its performance with that of a postoperative score and four clinical staging systems.

**Methods:**

Consecutive patients with surgically confirmed HCC who underwent preoperative EOB-MRI between July 2015 and November 2020 were retrospectively included. Two recurrence risk scores, one incorporating only preoperative variables and the other incorporating all preoperative and postoperative variables, were constructed via Cox regression models.

**Results:**

A total of 214 patients (derivation set, *n* = 150; test set, *n* = 64) were included. Six preoperative variables, namely tumor number, infiltrative appearance, corona enhancement, alpha-fetoprotein (AFP) level, aspartate aminotransferase (AST) level, and sex, were independently associated with recurrence. After adding postoperative features, microvascular invasion and tumor differentiation were additional significant variables in lieu of corona enhancement and AFP level. Using the above variables, the preoperative score achieved a C-index of 0.741 on the test set, which was comparable with that of the postoperative score (0.729; *p* = 0.235). The preoperative score yielded a larger time-dependent area under the receiver operating characteristic curve at 1 year (0.844) than three existing systems (0.734–0.742; *p* < 0.05 for all). Furthermore, the preoperative score stratified patients into two prognostically distinct risk strata with low and high risks of recurrence (*p* < 0.001).

**Conclusion:**

The preoperative score integrating EOB-MRI features, AFP and AST levels, and sex improves recurrence risk estimation in HCC.

**Key Points:**

*• The preoperative risk score incorporating three EOB-MRI findings, AFP and AST levels, and sex achieved comparable performance with that of the postoperative score for predicting recurrence after hepatectomy in patients with HCC.*

*• Two risk strata with low and high risks of recurrence were obtained based on the preoperative score.*

*• The preoperative score may help tailor pretreatment decision-making and facilitate candidate selection for adjuvant clinical trials.*

**Supplementary Information:**

The online version contains supplementary material available at 10.1007/s00330-022-08811-6.

## Introduction

Hepatocellular carcinoma (HCC) is the third leading cause of cancer-related death, with a growing incidence worldwide [1, 2]. Hepatic resection is widely accepted as a potentially curative treatment for patients with resectable HCC and well-preserved liver function [3, 4]. In concordance with the Barcelona Clinic Liver Cancer (BCLC) staging system, western guidelines recommend that liver resection is only eligible for very early and early HCC (BCLC stage 0 and A) [1, 2]. However, accumulated evidence suggests that surgical resection provides survival benefits for HCC patients with intermediate or advanced disease [5, 6]; therefore, guidelines from Asian areas have expanded the resection criteria, allowing selected individuals with intermediate and advanced HCC (BCLC stage B and C) to be considered for hepatectomy [7–11]. Unfortunately, tumor recurrence, including metastasis via primary tumor dissemination and de novo multicentric carcinogenesis, occurs in ~50–70% of patients within 5 years [3, 12].

Accurate risk estimation of recurrence is crucial for individualized treatment, management and surveillance strategies. Patients at high risk of recurrence following resection could benefit from adjuvant therapies. To date, several clinical staging systems, such as the BCLC system, American Joint Committee on Cancer (AJCC) tumor-node-metastasis (TNM) system, Hong Kong Liver Cancer (HKLC) system, and Japan Integrated Staging (JIS) score, constitute the cornerstones in prognostic stratification and treatment allocation for HCC [1, 2]. Nevertheless, it could be challenging to predict HCC recurrence according to the above systems because they are insufficient to profile the comprehensive landscape of tumor aggressiveness.

Gadoxetic acid–enhanced magnetic resonance imaging (EOB-MRI) has emerged as a first-line option for HCC diagnosis, staging, and surveillance. Recently, encouraging evidence has been proposed on the potential value of EOB-MRI for predicting outcomes in patients with HCC [13–19]. EOB-MRI features, such as arterial phase peritumoral enhancement [14, 16, 17], irregular tumor margin [18], peritumoral hypointensity on hepatobiliary phase (HBP) [17], satellite nodule [14, 17], and tumor size [14, 16, 19], have been reported to be predictive of postsurgical HCC recurrence. Despite the potential of these biomarkers, few studies have conducted a comprehensive assessment of tumor-related characteristics on EOB-MRI and proposed a noninvasive model for HCC recurrence with satisfactory predictive performance. Additionally, it is ambiguous whether prognostic tools integrating novel imaging biomarkers could compete with conventional clinical staging systems in terms of HCC recurrence prediction. To our knowledge, evidence comparing the prognostic value of preoperative EOB-MRI-based models with those of existing clinical staging systems remains scarce.

Therefore, we aimed to establish a recurrence risk score based on preoperative EOB-MRI and clinical parameters for HCC patients after hepatectomy and to compare its performance with that of a postoperative score and four clinical staging systems.

## Materials and methods

The institutional review board approved this single-center retrospective study and waived the requirement for informed consent.

### Patients

Between July 2015 and November 2020, consecutive adult (≥ 18 years) patients with pathologically confirmed HCC who underwent EOB-MRI before curative resection were recruited. The exclusion criteria were as follows: (a) any previous history of HCC treatment; (b) any co-malignancy other than HCC; (c) ruptured HCC; (d) presence of distant metastasis on preoperative work-up; (e) more than a 3-month interval between preoperative EOB-MRI and surgery; (f) unavailable laboratory or pathological data; (g) patients who underwent contemporary radiofrequency ablation or transarterial chemoembolization during the operation; (h) patients who died of postoperative complications within 2 weeks; and (i) loss to follow-up. For internally independent validation, eligible patients were randomly divided into a derivation set and a test set at a ratio of 7: 3.

Clinical (e.g., age, sex, and etiology), laboratory (e.g., aspartate aminotransferase [AST], alanine aminotransferase [ALT], and alpha-fetoprotein [AFP]), and histopathologic (e.g., microvascular invasion [MVI] and tumor differentiation) parameters were collected from electronic medical records. The calculation of albumin-bilirubin (ALBI) grade followed a previously described approach [20]. All patients were classified according to the BCLC system [1], 8th edition of the AJCC TNM system [21], HKLC system [10], and JIS score [22].

Tumor resectability was evaluated by the liver surgeons based on tumor burden, liver functional reserve, performance status, patient preference, and suggestions from the multidisciplinary team. All patients underwent curative resection (R0), defined as the complete removal of visible tumor tissue with a microscopically negative surgical margin.

### MRI technique

MRI examinations were performed with four 3.0-T systems (MAGNETOM Skyra, Siemens Healthineers; Discovery MR 750, GE Healthcare; SIGNA™ Architect, GE Healthcare; and SIGNA™ Premier, GE Healthcare) and a 1.5-T system (uMR588, United Imaging Healthcare). The MRI protocol included T2-weighted imaging, diffusion-weighted imaging, T1-weighted in-phase and opposed-phase imaging, and T1-weighted dynamic and HBP imaging using gadoxetic acid disodium (Primovist®, Bayer Pharma AG). Details of the MRI technique are provided in Supplementary A1 and Table S1.

### Image analysis

All MR images were independently reviewed by two abdominal radiologists (readers 1 and 2, with 7 and 5 years of experience in liver MR imaging, respectively) who were unaware of the clinical, laboratory, histopathologic, and follow-up information of the patients. Any discrepancy in imaging interpretation was resolved by a third radiologist (reader 3, with over 20 years of experience in liver MR imaging). Prior to the image analysis, each reader underwent a 1-month hands-on training with self-learning materials, including representative cases for each imaging feature and a brief lecture based on the Liver Imaging Reporting and Data System (LI-RADS) version 2018. The readers evaluated the following features for each patient: (a) tumor number; (b) tumor diameter; (c) presence or absence of all major, ancillary, LR-M and LR-TIV features as defined by LI-RADS version 2018 (except for threshold or subthreshold growth and ultrasound visibility as a discrete nodule due to lack of prior or concurrent ultrasound examinations); and (d) presence or absence of other imaging features that were related to tumor aggressiveness or outcome: internal artery, nonsmooth tumor margin, peritumoral hypointensity on HBP, tumor capsule (absent vs. complete vs. incomplete), liver cirrhosis, and bilobar involvement. For multifocal HCC, the radiologic features of the largest lesion were recorded for analysis. Definitions and representative images of EOB-MRI features are summarized in Table S2.

### Follow-up protocol

After surgery, the patients were followed up with serum AFP levels, liver function tests and dynamic imaging examinations (contrast-enhanced ultrasound, computed tomography or MRI) scheduled at 1 month after surgery, every 3 months for the first 2 years and then every 6 months thereafter. Tumor recurrence was diagnosed by imaging studies or pathologic examinations during follow-up after surgery. Recurrence-free survival (RFS) was defined as the time interval from surgery to the initial diagnosis of recurrence regardless of location. Patients alive and free of recurrence were censored at the end of the follow-up (August 20, 2021).

### Statistical analysis

Continuous variables were compared by Student’s t test or Mann-Whitney U test, whereas categorical variables were compared by chi-squared test or Fisher’s exact test, as appropriate.

Interobserver agreement of MRI findings was measured with Cohen’s κ coefficient for binary features, weighted κ coefficient for categorical features, and intraclass correlation coefficient for continuous variables.

#### Development and validation of preoperative and postoperative scores

Using the derivation set, two recurrence risk scores were constructed: (a) the preoperative score, which was developed based on preoperative clinical, laboratory and radiologic variables; and (b) the postoperative score, which was developed based on all preoperative variables as above plus postoperative pathologic features (MVI and tumor differentiation). To improve the clinical utility of the scores, continuous variables were converted into binary form according to normal ranges of laboratory indexes or clinical relevance.

While controlling for age and sex, univariable and multivariable Cox proportional hazards regression analyses were performed to identify significant risk factors for recurrence. Variables with *p* < 0.1 in the univariable analysis were included in the multivariable Cox regression model using a backward stepwise approach. Intervariable correlations were estimated by pairwise Spearman’s correlation analysis; when collinearity was encountered, predictors with the largest hazard ratio in univariable Cox regression analysis were kept for further analysis. The final models were formulated via the Akaike information criterion with fivefold cross-validation. Two recurrence risk scores were then generated based on the significant predictors in the final Cox models weighted by their regression coefficients (*β*). All scaled coefficients were rounded to the nearest integer, with the highest β coefficient assigned as 10 points.

Score discrimination was measured by Harrell’s concordance index (C-index) [23]. Calibration plots were used to depict the consistency between the predicted risk of recurrence and the observed risk [24]. Time-dependent receiver operating characteristic (tdROC) curve analysis was performed to quantify the predictive accuracy at various time points [25]. A decision curve analysis was conducted to determine the clinical utility and net benefit of the proposed scores [26].

#### Score comparison

The preoperative score was compared with the postoperative score, BCLC system, AJCC TNM system, HKLC system, and JIS score on both the derivation and test sets. Pairwise comparison of the C-index was performed using Student’s t test, while pairwise comparison of the time-dependent area under the receiver operating characteristic curve (tdAUC) was conducted with a previously described nonparametric approach [27].

#### Survival analysis

RFS was estimated by the Kaplan-Meier method and compared with the log-rank test. The frequencies of aggressive pathologic features (MVI and tumor differentiation) in the two preoperative recurrence risk strata were compared by chi-squared test.

All statistical analyses were performed with R software (version 3.5.1; The R Foundation for Statistical Computing) or SPSS software (version 22.0; IBM). The optimal cutoff points of the proposed scores for predicting recurrence were determined by X-tile software (version 3.6.1). Two-tailed *p* < 0.05 was considered statistically significant.

## Results

### Patient characteristics

**Fig. 1:**
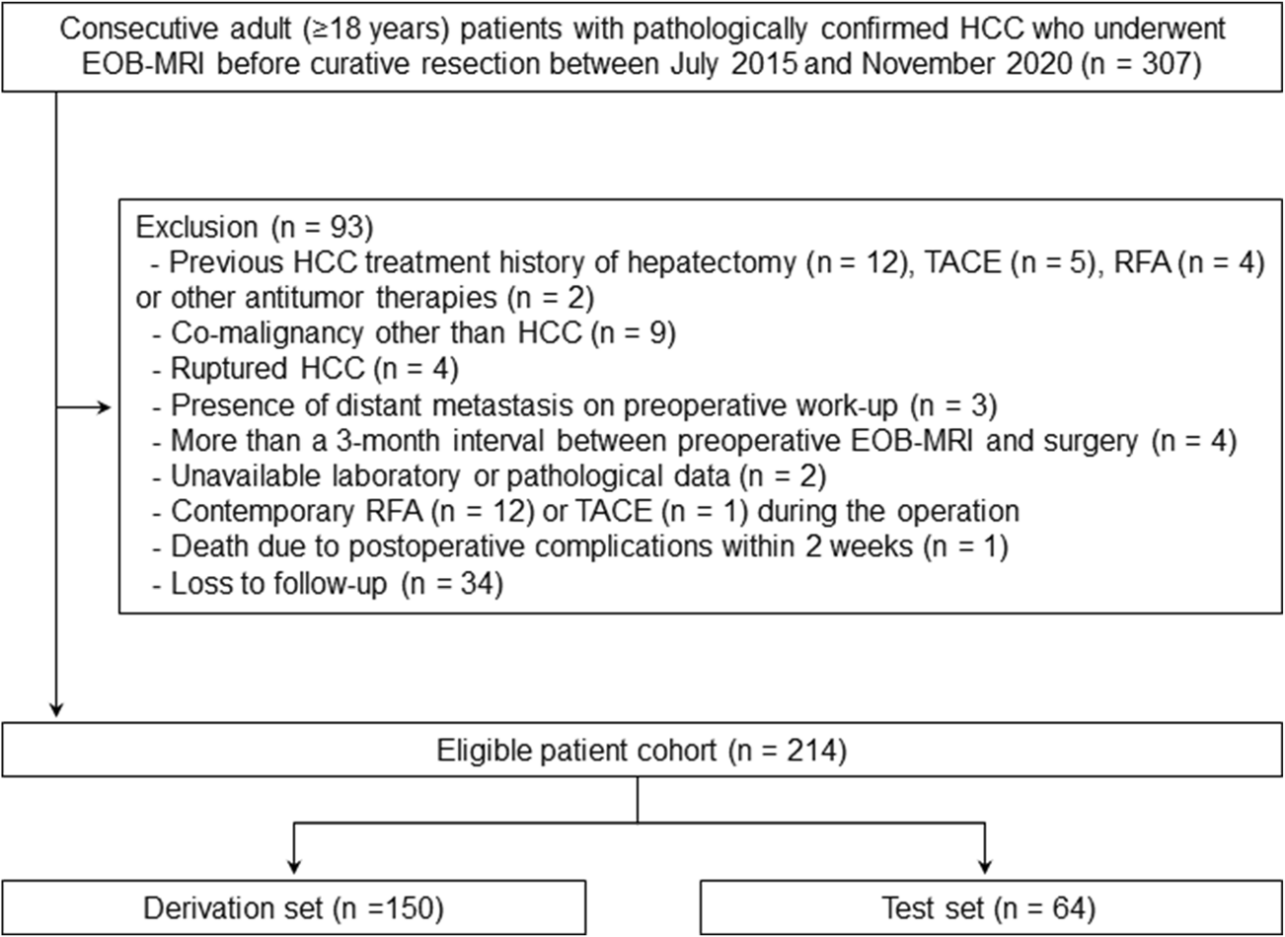
Flowchart of patient selection. EOB-MRI, gadoxetic acid–enhanced magnetic resonance imaging; HCC, hepatocellular carcinoma; RFA, radiofrequency ablation; TACE, transcatheter arterial chemoembolization

A total of 214 patients (median age, 53 years; interquartile range, 44–61 years; 181 men) were included in this study, among whom 150 and 64 patients were divided into the derivation and test sets, respectively (Fig. 1).

Patient characteristics are detailed in Table 1, clinical stages are summarized in Table 2, and frequencies of EOB-MRI features are shown in Table S3. No differences in clinical, radiologic, and histopathologic characteristics or follow-up information were detected between the derivation and test sets (*p* ≥ 0.05 for all). The median RFS was 29.3 months (95% confidence interval [CI]: 18.4 months, 51.6 months) for the derivation set and 40.0 months (95% CI: 14.8 months, not reached) for the test set (*p* = 0.845).

**Table 1:** Patient characteristics of the study cohort

Variable	Overall cohort (*n* = 214)	Derivation set (*n* = 150)	Test set (*n* = 64)	*p* value
Patient demographics				
Age, y	53.0 (44.0–61.0)	53.0 (44.0–61.3)	53.5 (46.3–60.0)	0.864
Sex (male/female)	181/33	130/20	51/13	0.196
Etiology				0.218
HBV	195 (91.1)	140 (93.3)	55 (85.9)	
HCV	3 (1.4)	2 (1.3)	1 (1.6)	
HBV and HCV	2 (0.9)	1 (0.7)	1 (1.6)	
Others	14 (6.5)	7 (4.7)	7 (10.9)	
Cirrhosis^†^	103 (48.8)	72 (48.3)	31 (50.0)	0.824
Laboratory index				
AST, IU/L				0.494
≤ 40	133 (62.1)	91 (60.7)	42 (65.6)	
> 40	81 (37.9)	59 (39.3)	22 (34.4)	
ALT, IU/L				0.223
≤ 50	155 (72.4)	105 (70.0)	50 (78.1)	
> 50	59 (27.6)	45 (30.0)	14 (21.9)	
TBIL, μmol/L				0.899
≤ 19	166 (77.6)	116 (77.3)	50 (78.1)	
>19	48 (22.4)	34 (22.7)	14 (21.9)	
ALB, g/L				0.081
≥ 40	164 (76.6)	110 (73.3)	54 (84.4)	
< 40	50 (23.4)	40 (26.7)	10 (15.6)	
PLT, x 10^9/L				0.430
≥ 100	163 (76.2)	112 (74.7)	51 (79.7)	
< 100	51 (23.8)	38 (25.3)	13 (20.3)	
PT, seconds				0.463
≤ 13	183 (85.5)	130 (86.7)	53 (82.8)	
> 13	31 (14.5)	20 (13.3)	11 (17.2)	
Child-Pugh grade				1.000
A	211 (98.6)	148 (98.7)	63 (98.4)	
B	3 (1.4)	2 (1.3)	1 (1.6)	
ALBI grade				0.130
1	134 (62.6)	88 (58.7)	46 (71.9)	
2	77 (36.0)	60 (40.0)	17 (26.6)	
3	3 (1.4)	2 (1.3)	1 (1.6)	
Serum AFP level, ng/mL				0.681
≤ 400	153 (71.5)	106 (70.7)	47 (73.4)	
> 400	61 (28.5)	44 (29.3)	17 (26.6)	
Tumor diameter, cm	4.1 (2.4–7.1)	4.0 (2.4–7.2)	4.4 (2.4–6.3)	0.956
Tumor number				0.181
1	145 (67.8)	107 (71.3)	38 (59.4)	
2 or 3	39 (18.2)	23 (15.3)	16 (25.0)	
> 3	30 (14.0)	20 (13.3)	10 (15.6)	
Histopathologic characteristic				
MVI	97 (45.3)	67 (44.7)	30 (46.9)	0.766
Tumor differentiation				0.515
Well	3 (1.4)	2 (1.3)	1 (1.6)	
Moderate	139 (65.0)	94 (62.7)	45 (70.3)	
Poor	72 (33.6)	54 (36.0)	18 (28.1)	
Surgical margin (R0)	214 (100.0)	150 (100.0)	64 (100.0)	…
Clinical outcome				
Recurrence	104 (48.6)	74 (49.3)	30 (46.9)	0.742
Recurrence-free survival^‡^				0.845
2-year rate, %	54.0 (62.0, 47.1)	53.0 (62.8, 44.7)	56.4 (71.2, 44.7)	
5-year rate, %	38.2 (48.0, 30.5)	35.9 (48.3, 26.7)	43.1 (61.5, 30.2)	
Median, months	30.8 (19.6, 45.2)	29.3 (18.4, 51.6)	40.0 (14.8, NA)	

Data are expressed as n (%) or median (interquartile range)

*HBV*, hepatitis B virus; *HCV*, hepatitis C virus; *AST*, aspartate aminotransferase; *ALT*, alanine aminotransferase; *TBIL*, total bilirubin; *ALB*, albumin; *PLT*, platelet; *PT*, prothrombin time; *ALBI*, albumin-bilirubin; *AFP*, alpha-fetoprotein; *MVI*, microvascular invasion; *NA*, not applicable

^†^Cirrhosis was diagnosed by the histopathologic examination and available in 211 (98.6%), 149 (99.3%), and 62 (96.9%) patients, respectively

^‡^Numbers in parentheses are the 95% confidence interval

**Table 2:** Clinical stages of all patients

Clinical stage	Overall cohort (*n* = 214)	Derivation set (*n* = 150)	Test set (*n* = 64)	*p* value
BCLC				0.050
0	31 (14.5)	23 (15.3)	8 (12.5)	
A	105 (49.1)	80 (53.3)	25 (39.1)	
B	33 (15.4)	17 (11.3)	16 (25.0)	
C	45 (21.0)	30 (20.0)	15 (23.4)	
HKLC				0.205
I	116 (54.2)	86 (57.3)	30 (46.9	
IIa	1 (0.5)	1 (0.7)	0 (0.0)	
IIb	45 (21.0)	30 (20.0)	15 (23.4)	
IIIa	1 (0.5)	1 (0.7)	0 (0.0)	
IIIb	49 (22.9)	32 (21.3)	17 (26.6)	
IVa	2 (0.9)	0 (0.0)	2 (3.1)	
JIS score				0.301
0	27 (12.6)	20 (13.3)	7 (10.9)	
1	102 (47.7)	76 (50.7)	26 (40.6)	
2	55 (25.7)	35 (23.3)	20 (31.3)	
3	29 (13.6)	19 (12.7)	10 (15.6)	
4	1 (0.5)	0 (0.0)	1 (1.6)	
AJCC TNM				0.142
IA	31 (14.5)	23 (15.3)	8 (12.5)	
IB	97 (45.3)	73 (48.7)	24 (37.5)	
II	37 (17.3)	26 (17.3)	11 (17.2)	
IIIA	35 (16.4)	21 (14.0)	14 (21.9)	
IIIB	12 (5.6)	7 (4.7)	5 (7.8)	
IVA	2 (0.9)	0 (0.0)	2 (3.1)	

Data are expressed as *n* (%)

*BCLC*, Barcelona Clinic Liver Cancer; *HKLC*, Hong Kong Liver Cancer; *JIS*, Japan Integrated Staging; *AJCC*, American Joint Committee on Cancer; *TNM*, tumor-node-metastasis

Of the 104 patients who experienced tumor recurrence, 63.5% (66/104) had exclusive intrahepatic recurrence, 2.9% (3/104) had exclusive extrahepatic recurrence, and 33.7% (35/104) had both intra- and extrahepatic recurrence.

### Score development on the derivation set

In the univariable analysis, 20 variables were significantly associated with HCC recurrence on the derivation set (Table S4). The multivariable analysis identified six significant parameters for inclusion in the preoperative Cox model: tumor number, infiltrative appearance, corona enhancement, AFP level > 400 ng/mL, AST level > 40 IU/L, and male sex (Table 3). For the postoperative Cox model, MVI and poor tumor differentiation were additional significant parameters included, whereas corona enhancement and AFP level > 400 ng/mL were excluded (Table 3).

**Table 3:** Multivariable Cox regression analysis of predictors for recurrence on the derivation set

Variable	Preoperative model					Postoperative model					Interobserver agreement (95 CI%)
	Hazard ratio (95% CI)	*p* value	β	Point		Hazard ratio (95% CI)	*p* value	β	Point		
Tumor number											0.653 (0.530, 0.777)
1	…	…	…	0		…	…	…	0		
2 or 3	2.615 (1.425, 4.799)	0.002	0.961	9		2.257 (1.217, 4.187)	0.010	0.814	8		
> 3	2.192 (1.098, 4.375)	0.026	0.785	7		2.136 (1.038, 4.395)	0.039	0.759	7		
Infiltrative appearance	2.152 (1.046, 4.430)	0.037	0.767	7		2.179 (1.091, 4.354)	0.027	0.779	7		0.622 (0.474, 0.771)
Corona enhancement	1.132 (0.660, 1.942)	0.653	0.124	1		NA	NA	NA	NA		0.326 (0.174, 0.478)
AFP (> 400 ng/mL)	1.811 (1.073, 3.058)	0.026	0.594	5		NA	NA	NA	NA		NA
AST (> 40 IU/L)	2.960 (1.829, 4.793)	< 0.001	1.085	10		2.901 (1.799, 4.680)	< 0.001	1.065	10		NA
Sex (male)	1.906 (0.857, 4.238)	0.114	0.645	6		2.522 (1.106, 5.750)	0.028	0.925	9		NA
MVI	NA	NA	NA	NA		1.959 (1.155, 3.320)	0.013	0.672	6		NA
Tumor differentiation (poor)	NA	NA	NA	NA		1.586 (0.978, 2.572)	0.061	0.462	4		NA

*AFP*, alpha-fetoprotein; *AST*, aspartate aminotransferase; *MVI*, microvascular invasion; *CI*, confidence interval; *NA*, not applicable

**Fig. 2:**
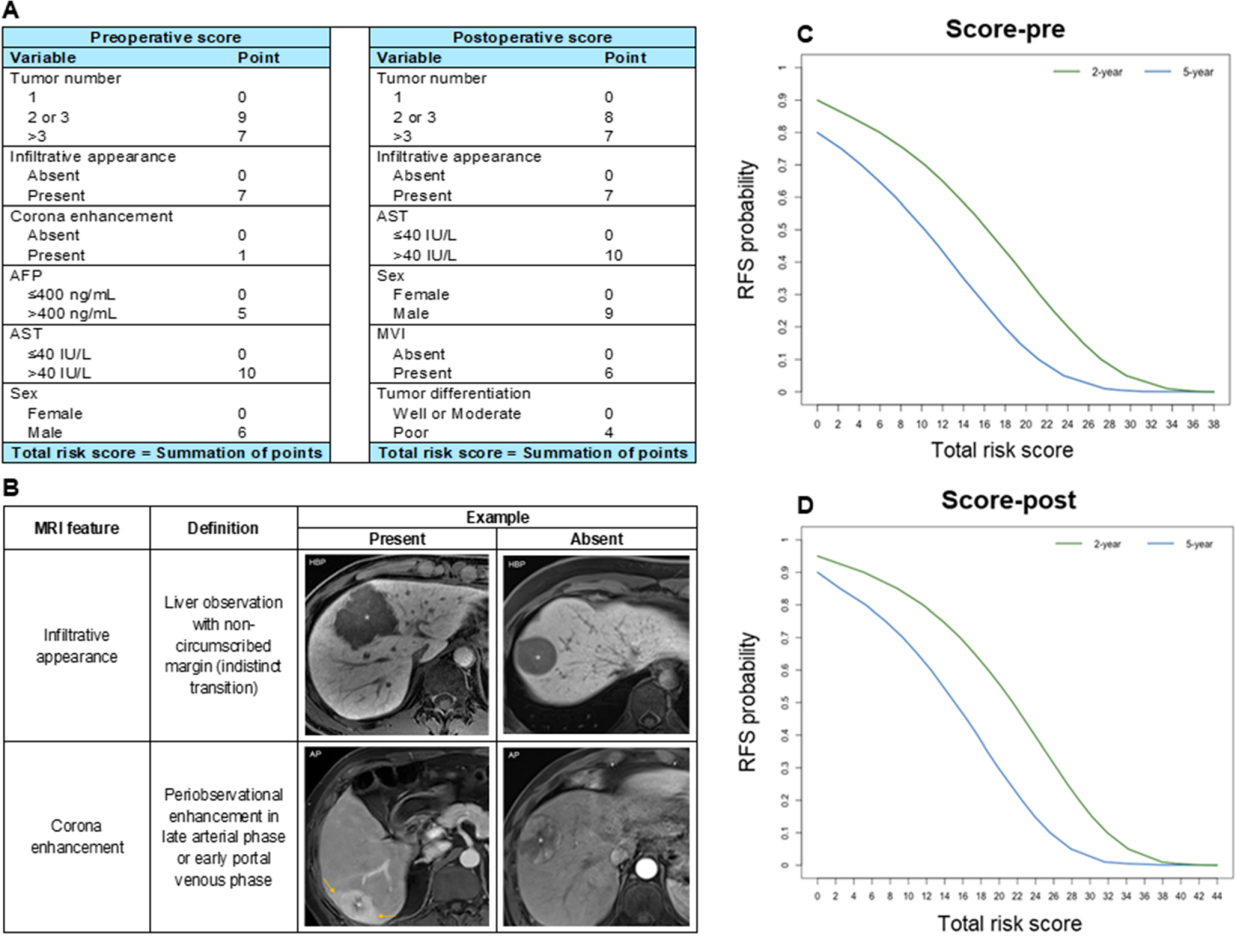
**A** The preoperative and postoperative recurrence risk scores for patients with hepatocellular carcinoma after resection; **B** Definitions and representative images of MRI features included in the established scores; **C** Probability of 2- and 5-year recurrence-free survival according to the preoperative total risk score; and **D** Probability of 2- and 5-year recurrence-free survival according to the postoperative total risk score. AFP, alpha-fetoprotein; AST, aspartate aminotransferase; MVI, microvascular invasion; MRI, magnetic resonance imaging; AP, arterial phase; HBP, hepatobiliary phase; RFS, recurrence-free survival

The preoperative and postoperative models that incorporated the corresponding predictors were constructed. Two recurrence risk scores based on the above models were generated and are illustrated in Fig. 2. The total risk score for recurrence prediction was calculated by adding the individual points of each variable, which ranged from 0 to 38 points for the preoperative score and from 0 to 44 points for the postoperative score.

### Score assessment on the derivation set

The preoperative recurrence risk score achieved a C-index of 0.756 (95% CI: 0.695, 0.817), which was comparable with that of the postoperative score (0.770 [95% CI: 0.709, 0.831]; *p* = 0.863). Additionally, there were no statistically significant differences in C-indexes between the preoperative score and conventional staging systems (*p* > 0.05 for all) (Table S5).

**Fig. 3:**
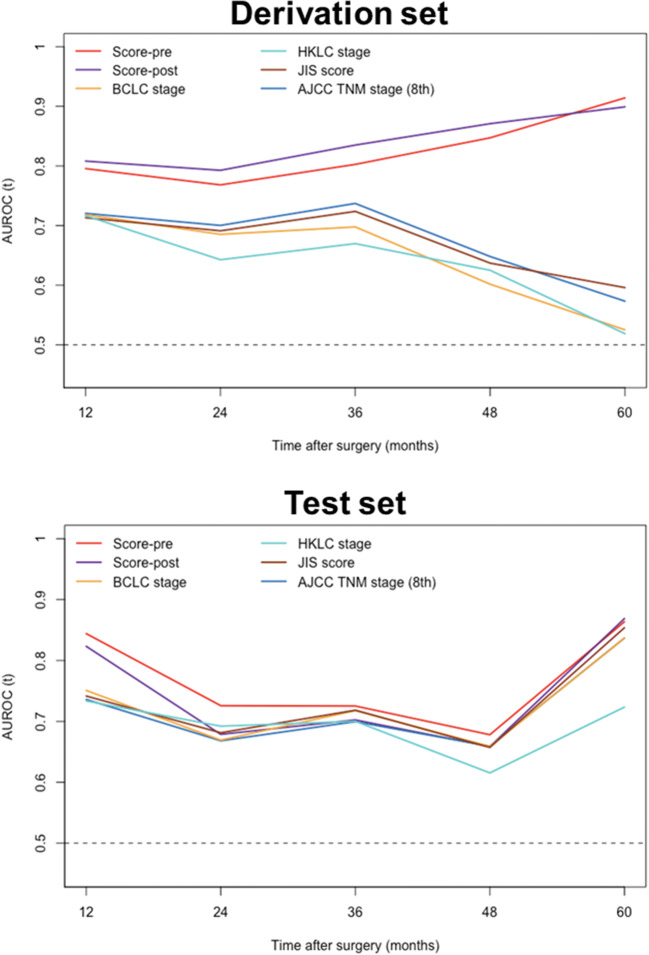
Time-dependent areas under the receiver operating characteristic curve from 12 to 60 months for proposed scores and staging systems. AJCC, American Joint Committee on Cancer; AUROC, areas under the receiver operating characteristic; BCLC, Barcelona Clinic Liver Cancer; HKLC, Hong Kong Liver Cancer; JIS, Japan Integrated Staging; TNM, tumor-node-metastasis

Calibration plots for the preoperative and postoperative scores showed an overall good consistency between the predicted probabilities and the observed outcome on the derivation set (Fig. S1). Using tdROC curve analysis, the preoperative and postoperative scores exhibited similar predictive accuracies at various time points (*p* > 0.05 for all) (Fig. 3; Table S5). In addition, the preoperative score yielded superior accuracies when compared to existing staging systems at various time points on the derivation set (Fig. 3; Table S5). Decision curves revealed that the preoperative score provided a larger net benefit than conventional staging systems on the derivation set (Fig. S2).

### Score validation on the test set

Likewise, the preoperative and postoperative scores exhibited comparable discriminatory performance outcomes on the test set, with C-indexes of 0.741 (95% CI: 0.664, 0.818) and 0.729 (95% CI: 0.646, 0.812), respectively (*p* = 0.235). However, no difference in C-indexes was observed when comparing the preoperative score with other clinical staging systems (*p* > 0.05 for all) (Table S5).

Calibration plots for the preoperative and postoperative scores yielded an overall good agreement between the predicted probabilities and the actual outcome on the test set (Fig. S1). In terms of the tdROC curve analysis, the preoperative and postoperative scores demonstrated similar tdAUCs at various time points on the test set (*p* > 0.05 for all) (Fig. 3; Table S5). Moreover, the preoperative score displayed a significantly higher tdAUC than that of three existing systems (HKLC stage, JIS score, and AJCC TNM stage) at 1 year (*p* < 0.05 for all) (Fig. 3; Table S5). Regarding the clinical utility, the preoperative score showed an overall larger net benefit than the postoperative score and existing staging systems on the test set (Fig. S2).

### Recurrence risk stratification according to the preoperative score

**Fig. 4:**
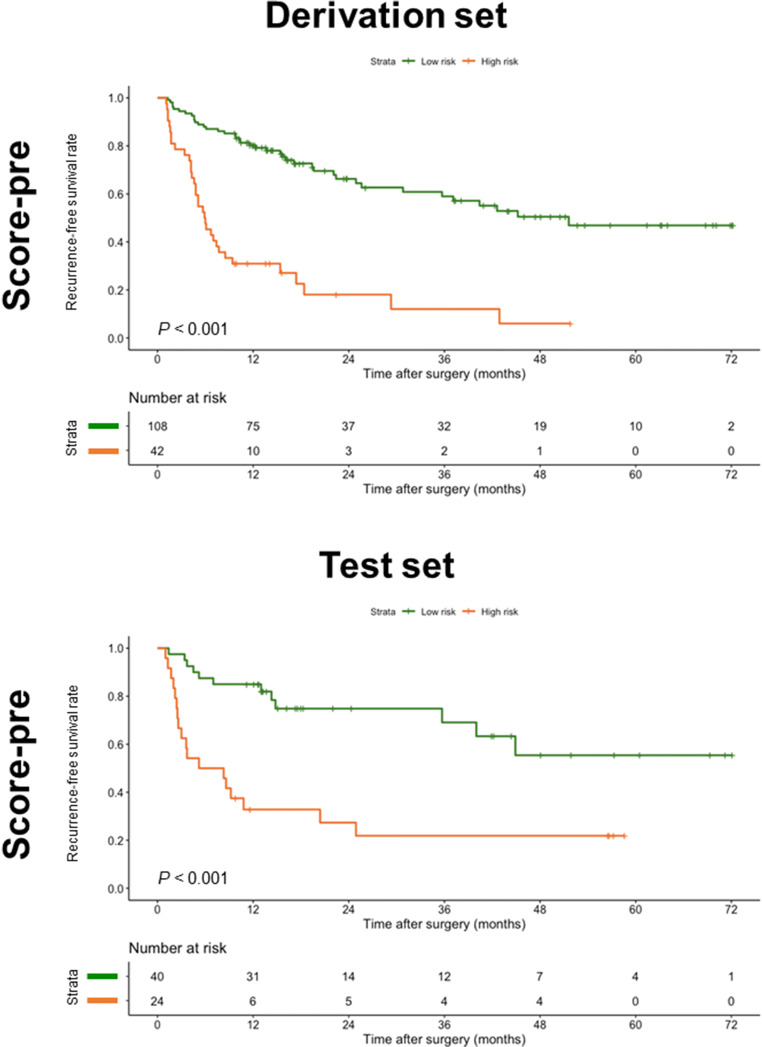
Recurrence-free survival curves according to two risk strata defined by the preoperative score

Using 17 as the cutoff for the preoperative score on the derivation set, the patients were stratified into two prognostically distinct groups: low-risk and high-risk groups (median RFS, 51.6 months vs. 6.0 months; *p* < 0.001). The 2- and 5-year RFS rates were 66.3% and 46.9% for low-risk patients, and 18.1% and 6.0% for high-risk patients, respectively. Based on this cutoff score, the preoperative score partitioned the patients into two distinct prognostic strata on the test set (median RFS of the low-risk and high-risk groups, not reached vs. 6.8 months; *p* < 0.001) (Fig. 4; Table S6).

The frequencies of MVI (derivation set, 34.3% vs. 71.4%, *p* < 0.001; test set, 27.5% vs. 79.2%, *p* < 0.001) and poor tumor differentiation (derivation set, 30.6% vs. 50.0%, *p* = 0.026; test set, 17.5% vs. 45.8%, *p* = 0.015) were significantly different between the low-risk and high-risk groups (Table S7).

## Discussion

In the present study, we developed and validated two prognostic scores based on readily accessible preoperative and postoperative clinical, EOB-MRI, and pathologic parameters for predicting HCC recurrence after curative resection. Intriguingly, the preoperative score exhibited satisfactory prognostic performance comparable to that of the postoperative score, offering a potential noninvasive and reliable approach for preoperative individualized recurrence risk estimation. Moreover, the preoperative score yielded superior predictive performance to currently adopted clinical staging systems for HCC recurrence prediction. This tool can be used to individualize HCC management based on recurrence risk stratification. By identifying patients at high risk of recurrence prior to treatment, the proposed score may be instrumental in refining treatment protocols (e.g., performing more aggressive surgery or expanding ablation zones and considering intraarterial or systemic treatment in the adjuvant or neoadjuvant setting), tailoring follow-up schedules with more intensive surveillance and sensitive techniques (e.g., EOB-MRI), and selecting candidates for clinical trials of combination regimens. Furthermore, the proposed score consisting of simplified point scales may facilitate the bedside calculation of the scores and enhance patient counseling.

To our knowledge, this is the first study to directly compare the preoperative EOB-MRI-based score with four conventional staging systems for postoperative HCC recurrence prediction. In the present study, the preoperative score achieved an overall better predictive accuracy and a larger net benefit than the existing staging systems. Despite the slight advantages, novel clinical-radiologic biomarkers showed promise to improve the risk estimation of HCC recurrence to compensate for traditional staging systems. Further investigation is needed to clarify the incremental value of novel imaging biomarkers to existing staging systems. However, the incorporation of semantic features alone is probably insufficient to optimize the prognostic scoring. More robust imaging biomarkers, such as semiquantitative and quantitative parameters, should be explored.

Notably, in our study, the four clinical staging systems yielded a higher discriminatory performance (C-indexes: 0.712–0.762) than that of previous studies for predicting HCC recurrence (C-indexes: 0.510–0.730) [28–31]. We speculate that the larger proportion of intermediate and advanced (BCLC stage B and C) HCC patients (36.4%; 78/214) in our study cohort might be responsible for this discrepancy. Although patients with very early or early HCC (BCLC stage 0 and A) are perceived as optimal candidates for resection [1], accumulating evidence has shown that surgical resection can benefit selected patients with intermediate and advanced HCC (e.g., patients with local portal vein thrombosis) [5, 6, 32–35]. Consequently, Asian guidelines recommend liver resection as a treatment option for carefully selected individuals with BCLC stage B and C HCC [7–11]. Despite representing a marked deviation from several Western guidelines, the study population of the current study shadowed the real-world clinical routine of large tertiary care centers in China, where up to 5.4–26% of surgical patients had advanced-stage tumors [36]. In this context, multidisciplinary discussion is essential to balance surgical benefits with potential adverse effects.

MVI and tumor differentiation have been identified as independent risk factors for HCC recurrence, as demonstrated by our study and prior work [37–38]. It is worthwhile to note that the frequencies of MVI and poor tumor differentiation increased significantly from the low-risk group to the high-risk group based on the preoperative recurrence risk stratification. These results shed light on the potential histopathologic mechanisms underlying the preoperative score in this study, revealing the radiologic-pathologic linkages.

Corona enhancement was depicted as a high-risk area for metastatic satellites associated with local recurrence in hypervascular, progressed HCC [39]. Our results recapitulated the findings of previous studies, showing that corona enhancement was predictive of HCC recurrence after surgical resection [14, 16, 17]. To improve the curative efficacy and decrease the recurrence risk, some investigators recommended a wider resection margin or ablation zone for removing the corona enhancement area [39, 40]. Additionally, infiltrative appearance was included in our recurrence risk scores, which could be explained by the fact that infiltrative appearance has been associated with a more aggressive phenotype of HCC [41, 42].

Interestingly, AST level > 40 IU/L was the independent variable most closely related to recurrence in our study, as previously reported [43, 44]. Although almost all patients in our study cohort (98.6%; 211/214) were classified as Child-Pugh grade A, our models still strongly relied on this laboratory index. This underscores the usefulness of such serum markers in individualized prognostication, even among patients with well-preserved liver function. However, the exact mechanisms underlying increased AST levels in tumor recurrence are not well understood. In addition, AFP level > 400 ng/mL was an independent predictor of HCC recurrence in our study, in accordance with the findings of previous studies [17, 45]. Further investigation is needed to decipher the underlying biologic mechanisms of these linkages.

This study has several limitations. First, due to its retrospective design, potential selection bias may exist. Second, it was a single-center study, and expanding our results to other medical centers is needed to confirm their reliability and reproducibility. Third, a large proportion of patients had hepatitis B virus-related HCC. Therefore, further validation of our results in populations with other etiologies will be needed to check for generalizability. Finally, the association of the minimal resection safety margin with recurrence was not investigated because detailed data on the surgical margin were unavailable owing to the retrospective design. Nonetheless, all specimens presented negative margins (R0) at postsurgical pathological examinations, indicating that the resected livers were free of residual tumor cells. However, minimal resection safety margin is a crucial prognostic factor associated with HCC recurrence. In particular, an adequate resection margin may help to improve the chance of micrometastasis clearance, thereby preventing tumor recurrence. Although the evaluation of the minimal resection safety margin is beyond the scope of the present study, it is certainly a critical issue that warrants detailed analysis in future research.

In conclusion, the preoperative score integrating EOB-MRI features, serum AFP and AST levels, and sex allowed accurate recurrence prediction in HCC, with similar performance to that of the postoperative assessment. Moreover, the preoperative score yielded slight advantages over existing staging systems for HCC recurrence prediction. Further studies are needed to investigate the incremental value of quantitative imaging biomarkers to conventional HCC staging systems.

## Supplementary information

ESM 1 (DOCX 9388 kb)

## Abbreviations

AFP: Alpha-fetoprotein
AJCC: American Joint Committee on Cancer
ALBI: Albumin-bilirubin
ALT: Alanine aminotransferase
AST: Aspartate aminotransferase
BCLC: Barcelona Clinic Liver Cancer
CI: Confidence interval
C-index: Concordance index
EOB-MRI: Gadoxetic acid–enhanced magnetic resonance imaging
HBP: Hepatobiliary phase
HCC: Hepatocellular carcinoma
HKLC: Hong Kong Liver Cancer
JIS: Japan Integrated Staging
LI-RADS: Liver Imaging Reporting and Data System
MVI: Microvascular invasion
RFS: Recurrence-free survival
tdAUC: Time-dependent area under the receiver operating characteristic curve
tdROC: Time-dependent receiver operating characteristic
TNM: Tumor-node-metastasis

## Declarations

### Guarantor

The scientific guarantor of this publication is Bin Song, M.D.

### Statistics and biometry

One co-author (Yuanan Wu, Big Data Research Center, University of Electronic Science and Technology of China, Chengdu, Sichuan, China) has significant statistical expertise.

### Methodology

• retrospective

• diagnostic or prognostic study

• performed at one institution
