# Supplementary material for: Comparison of a preoperative MR-based recurrence risk score versus the postoperative score and four clinical staging systems in hepatocellular carcinoma: a retrospective cohort study
Source: Eur Radiol. 2022 May 13;32(11):7578–89. doi: 10.1007/s00330-022-08811-6 (PMC9668764; doi:10.1007/s00330-022-08811-6)
Supplement: Supplementary file 1 — (DOCX 9388 kb) [file 330_2022_8811_MOESM1_ESM.docx]

**Supplementary Material**

**Table of Contents**

**Supplementary A1 MRI technique 1**

**Table S1 MRI sequences and parameters2**

**Table S2 Definitions and representative images of EOB-MRI features 4**

**Table S3 Frequencies of EOB-MRI features 14**

**Table S4 Univariable Cox regression analysis of predictors for recurrence on the derivation set 16**

**Table S5 Prognostic performance of the preoperative score compared with the postoperative score and four clinical staging systems 18**

**Table S6 Median RFS, 2- and 5-year RFS rates, and hazard ratio according to each risk group as defined by the preoperative score 20**

**Table S7 Aggressive pathologic features in the preoperative recurrence risk strata 21**

**Fig. S1 Calibration plots for 2- and 5-year RFS obtained by the preoperative and postoperative scores 22**

**Fig. S2 Decision curves for 2- and 5-year RFS obtained by the preoperative and postoperative scores and existing staging systems 23**

**Supplementary A1**

**MRI** **technique**

MRI examinations were performed with four 3.0-T systems (MAGNETOM Skyra, Siemens Healthineers; Discovery MR 750, GE Healthcare; SIGNA™ Architect, GE Healthcare; and SIGNA™ Premier, GE Healthcare) and a 1.5-T system (uMR588, United Imaging Healthcare). The MRI sequences included T2-weighted two-dimensional fast spin echo imaging, diffusion-weighted imaging (b values: 0, 50, 500, 800, 1000, and 1200 s/mm^2^ [Siemens MAGNETOM Skyra]; 0, 200, 800, and 1000 s/mm^2^ [GE Discovery MR 750]; 0, 50, and 800 s/mm^2^ [GE SIGNA™ Architect 3.0 Tesla]; 50 and 1000 s/mm^2^ [GE SIGNA™ Premier 3.0 Tesla]; and 0, 50, and 1000 s/mm^2^ [uMR588 1.5 Tesla]) with apparent diffusion coefficient (ADC) maps, T1-weighted dual gradient-echo in- and opposed-phase imaging, and dynamic T1- weighted three-dimensional gradient-echo imaging before and after injection of gadoxetic acid disodium (Primovist®, Bayer Pharma AG) in the late arterial phase, portal venous phase (60 s), transitional phase (3 minutes), and hepatobiliary phase (20 minutes). The arterial phase images were determined either by the acquisition triggered 7 s after the arrival of the contrast bolus in the celiac trunk or by a multiple arterial phase (MAP) imaging technique. Specifically, the MAP images were acquired with an 18 s breath hold 20 s following the contrast agent injection and further reconstructed with a temporal resolution of 3 s. For dynamic imaging, the contrast agent was injected at a rate of 1-2 ml/s for a total dose of 0.025 mmol/kg body weight, followed by 20-30 ml of 0.9% saline flush. Details of the MRI sequences and parameters are shown in **Table S1.**

**Table S1** MRI sequences and parameters

| Sequence | T1-weighted IP and OP imaging | Dynamic T1-weighted 3D GRE | T2-weighted 2D FSE | Diffusion-weighted imaging^†^ |
| --- | --- | --- | --- | --- |
| Siemens MAGNETOM Skyra (18-channel body array coil) | | | | |
| Repetition time (ms) | 81 | 3.95 | 2160 | 5600 |
| Echo time (ms) | 2.72/1.4 | 1.92 | 100 | 68 |
| Flip angle (°) | 70 | 9 | 160 | 90 |
| Section thickness (mm) | 6 | 2.5 | 6 | 6 |
| Spacing (mm) | 1.8 | - | 1.8 | 1.8 |
| Matrix size | 352×286 | 352×256 | 320×288 | 100×76 |
| Field of view (mm^2^) | 400×325 | 400×296 | 433×433 | 380×289 |
| Acquisition time (s) | 24 | 14 | 36 | 233 |
| Fat suppression | No | Yes | Yes | Yes |
| GE Discovery MR 750 (16-channel phased-array torsor coil) | | | | |
| Repetition time (ms) | 150 | 4.1 | 6315 | 9230 |
| Echo time (ms) | 2.5/1.3 | 1.9 | 78 | Minimum |
| Flip angle (°) | 70 | 15 | 111 | 90 |
| Section thickness (mm) | 6 | 2 | 6 | 6 |
| Spacing (mm) | 2 | - | 2 | 2 |
| Matrix size | 288×192 | 512×512 | 288×244 | 128 × 128 |
| Field of view (mm^2^) | 420×420 | 380× 300 | 360×280 | 360× 380 |
| Acquisition time (s) | 31 | 15 | RG | RG |
| Fat suppression | No | Yes | Yes | Yes |
| GE SIGNA™ Architect 3.0 Tesla (30-channel body anterior coil) | | | | |
| Repetition time (ms) | 233.8 | 3.9 | 2400 | 5000 |
| Echo time (ms) | 2.3/1.1 | 1.7 | 85 | Minimum |
| Flip angle (°) | 55 | 15 | 111 | 90 |
| Section thickness (mm) | 7 | 3 | 7 | 7 |
| Spacing (mm) | 2 | - | 2 | 2 |
| Matrix size | 160×288 | 320×240 | 320×192 | 160×128 |
| Field of view (mm^2^) | 380×323 | 380×380 | 380×304 | 380×342 |
| Acquisition time (s) | 18 | 15 | 34 | RG |
| Fat suppression | No | Yes | Yes | Yes |
| GE SIGNA™ Premier 3.0 Tesla (30-channel body anterior coil) | | | | |
| Repetition time (ms) | 146.8 | 3.2 | 2200 | 5000 |
| Echo time (ms) | 2.3/1.1 | 1.4 | 85 | Minimum |
| Flip angle (°) | 55 | 15 | 111 | 90 |
| Section thickness (mm) | 7 | 2.4 | 7 | 7 |
| Spacing (mm) | 2 | - | 2 | 2 |
| Matrix size | 320×192 | 320×240 | 320×224 | 120 × 240 |
| Field of view (mm^2^) | 342×380 | 380× 380 | 304×380 | 380× 380 |
| Acquisition time (s) | 16 | 15 | 47 | RG |

| Fat suppression | No | Yes | Yes | Yes |
| --- | --- | --- | --- | --- |
| uMR588 1.5 Tesla (6-channel body anterior coil) | | | | |
| Repetition time (ms) | 117.6 | 4.2 | 2600 | 3350 |
| Echo time (ms) | 2.27 | 1.88 | 99.2 | 77 |
| Flip angle (°) | 60 | 10 | 90 | 90 |
| Section thickness (mm) | 6.5 | 2.5 | 6.5 | 6.5 |
| Spacing (mm) | 1.3 | - | 1.5 | 10 |
| Matrix size | 256×174 | 256×154 | 256×168 | 128×92 |
| Field of view (mm^2^) | 320×400 | 255×400 | 427×320 | 320×400 |
| Acquisition time (s) | 29 | 13 | 39 | RG |
| Fat suppression | No | Yes | Yes | Yes |

MRI, magnetic resonance imaging; IP, in-phase; OP, opposed-phase; 3D, three-dimensional; GRE, gradient recall echo; 2D, two-dimensional; FSE, fast spin-echo; RG, respiratory gating.

^†^Images were acquired under free breath.

**Table S2** Definitions and representative images of EOB-MRI features

| **MRI feature**^†^ | **Definition** | **Example** | | | |
| --- | --- | --- | --- | --- | --- |
| **Tumor number**^§^ | Number of definite intrahepatic HCC lesions with characteristic enhancement pattern [1-3] | - | | | |
| **Tumor diameter or Size** | Largest outer-edge-to-outer-edge dimension of a liver observation [4] | 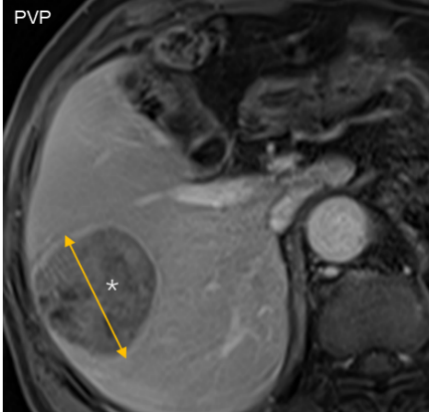 | | 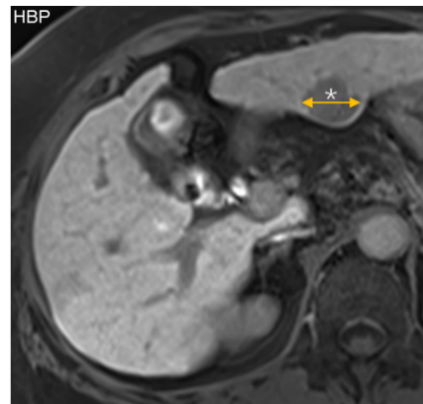 | |
|  |  | Include “capsule” in measurement | | Pick phase, sequence, plane in which margins are clearest | |
| **Nonrim arterial phase hyperenhancement** | Nonrim-like enhancement of the liver observation in arterial phase unequivocally greater in whole or in part than liver [4] | 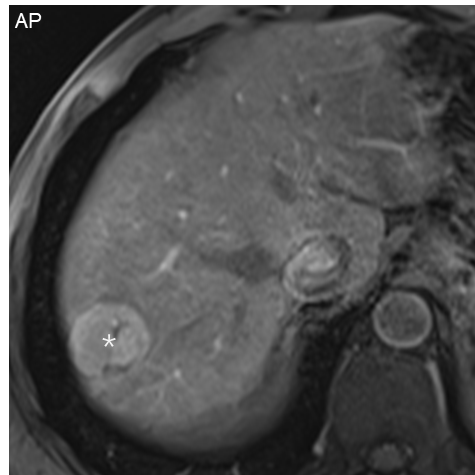 | | 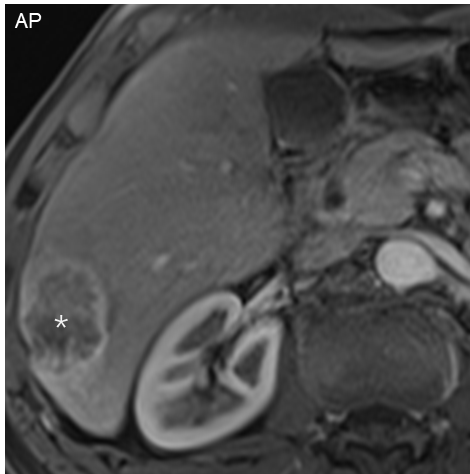 | |
|  |  | Present | | Absent | |
| **Nonperipheral "washout"** | Nonperipheral visually assessed temporal reduction in enhancement of the liver observation in whole or in part relative to composite liver tissue from earlier to later phase resulting in hypoenhancement in the extracellular phase [4] | 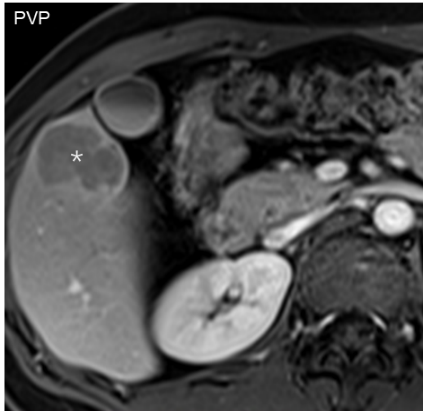 | | 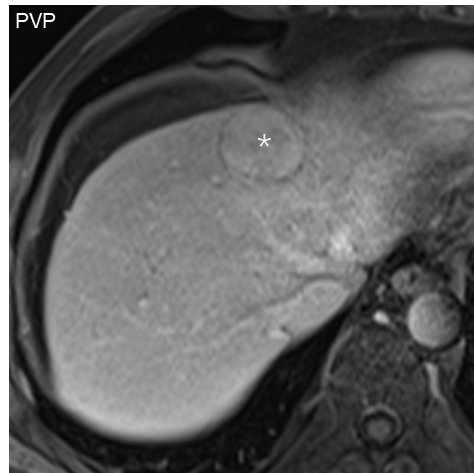 | |
|  |  | Present | | Absent | |
| **Enhancing "capsule"** | Smooth, uniform, sharp border around most or all of a liver observation, unequivocally thicker or more conspicuous than fibrotic tissue around background nodules, and visible as enhancing rim in portal venous phase or transitional phase [4] | 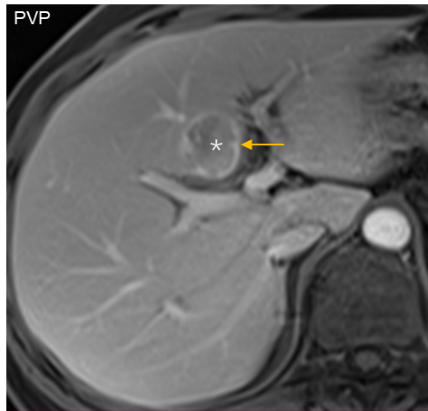 | | 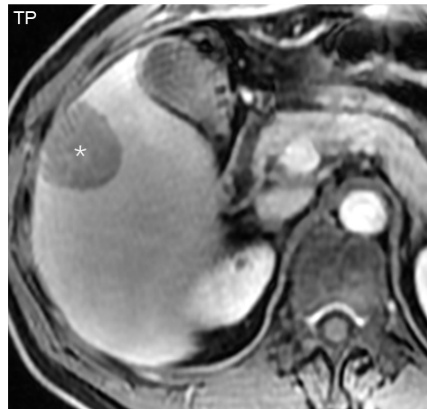 | |
|  |  | Present | | Absent | |
| **Corona enhancement** | Periobservational enhancement in late arterial phase or early portal venous phase attributable to venous drainage from tumor [4] | 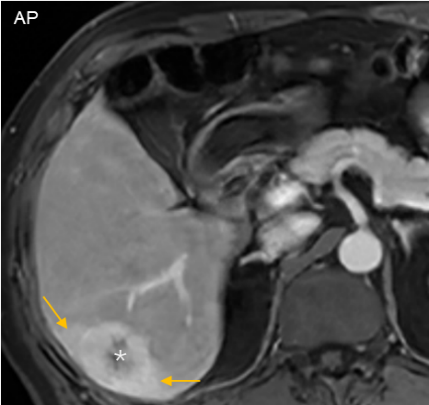 | | 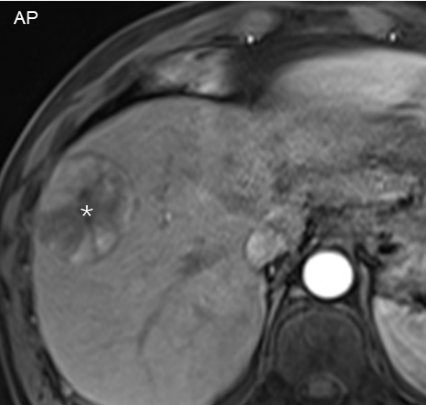 | |
|  |  | Present | | Absent | |
| **Fat sparing in solid mass** | Relative paucity of fat in solid mass relative to steatotic liver OR in inner nodule relative to steatotic outer nodule [4] | 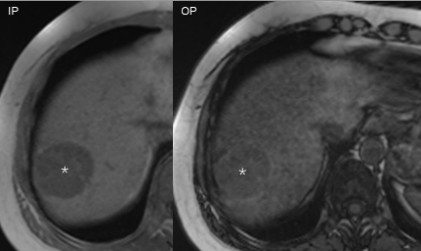 | | 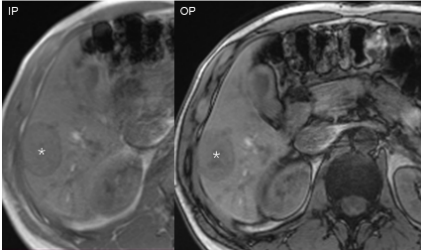 | |
|  |  | Present | | Absent | |
| **Restricted diffusion**^‡^ | Signal intensity of the liver observation on diffusion-weighted imaging, not attributable solely to T2 shine-through, unequivocally higher than liver and/or apparent diffusion coefficient unequivocally lower than liver [4] | 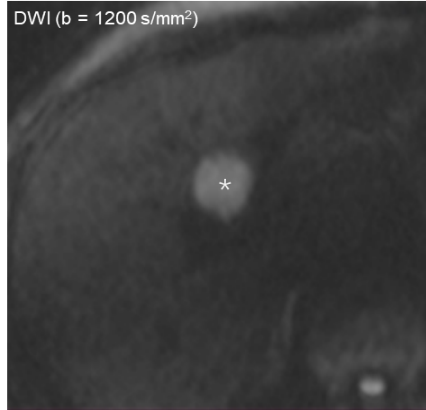 | | - | |
|  |  | Present | | Absent | |
| **Mild-moderate T2 hyperintensity** | Signal intensity of the liver observation on T2-weighted imaging mildly or moderately higher than liver and similar to or less than non-iron-overloaded spleen [4] | 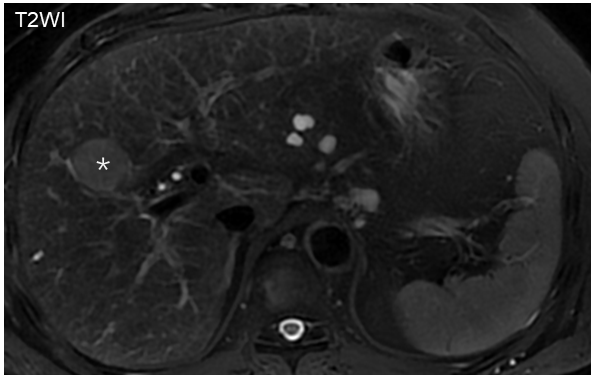 | | 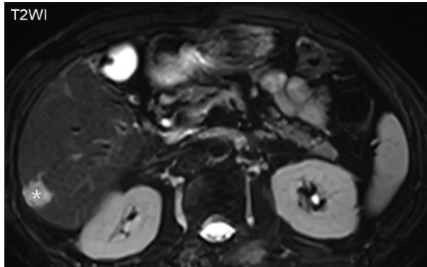 | |
|  |  | Present | | Absent | |
| **Iron sparing in solid mass** | Paucity of iron in solid mass relative to iron-overloaded liver OR in inner nodule relative to siderotic outer nodule [4] | 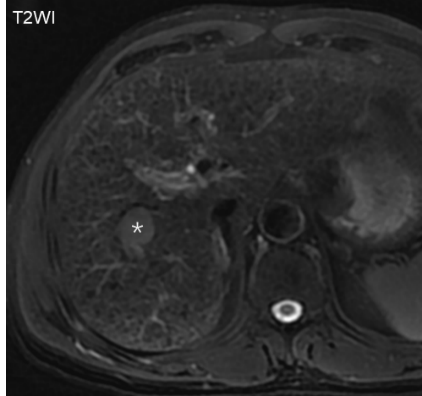 | | 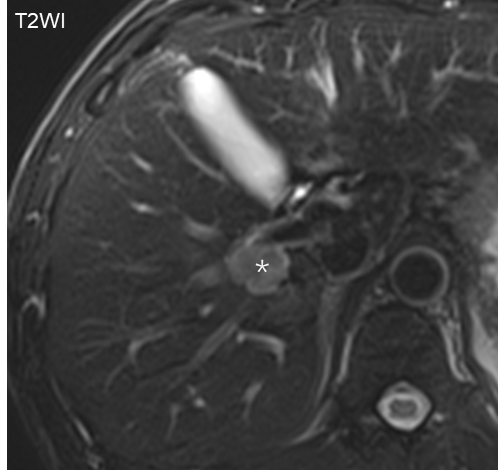 | |
|  |  | Present | | Absent | |
| **Transitional phase hypointensity** | Signal intensity of the liver observation in the transitional phase unequivocally less, in whole or in part, than liver [4] | 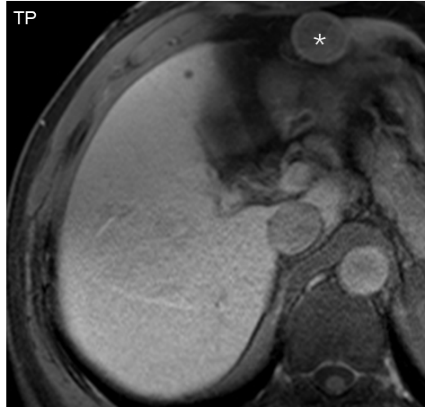 | | 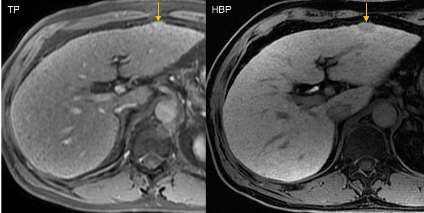 | |
|  |  | Present | | Absent | |
| **Hepatobiliary phase hypointensity** | Signal intensity of the liver observation in the hepatobiliary phase unequivocally less, in whole or in part, than liver [4] | 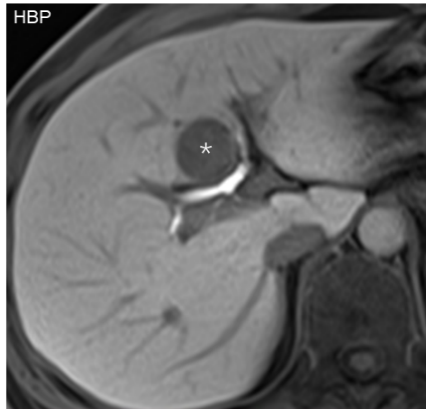 | | 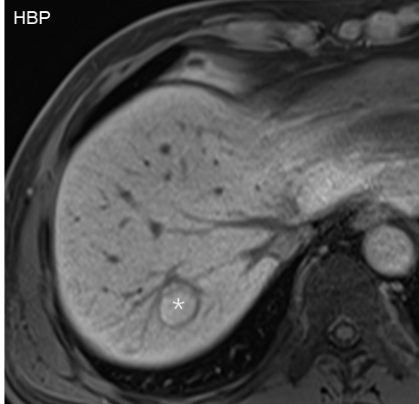 | |
|  |  | Present | | Absent | |
| **Non-enhancing "capsule"** | Capsule appearance not visible as an enhancing rim [4] | 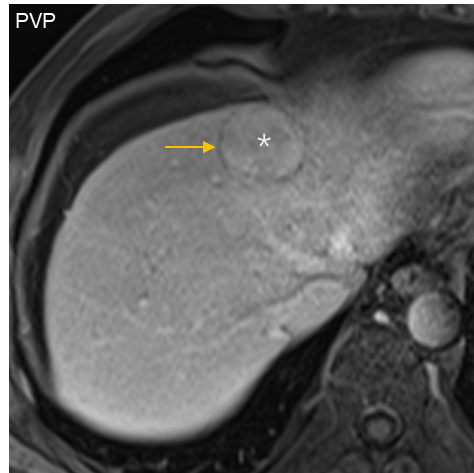 | | 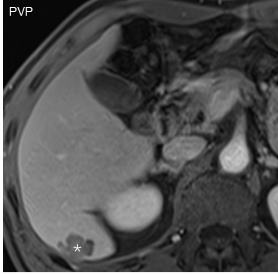 | |
|  |  | Present | | Absent | |
| **Nodule-in-nodule architecture** | Presence of smaller inner nodule within and having different imaging features than larger outer nodule [4] | 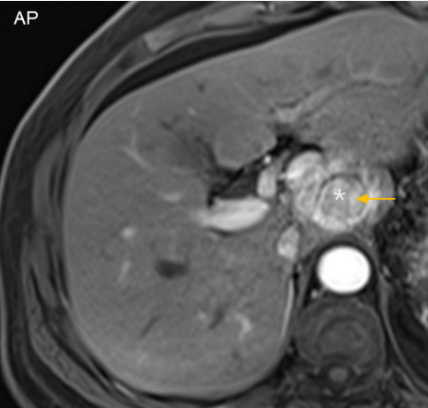 | | 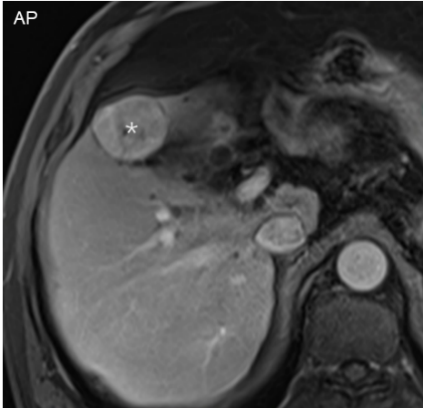 | |
|  |  | Present | | Absent | |
| **Mosaic architecture** | Presence of randomly distributed internal nodules or compartments, usually with different imaging features [4] | 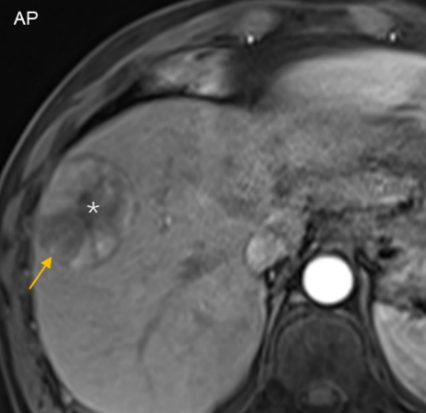 | | 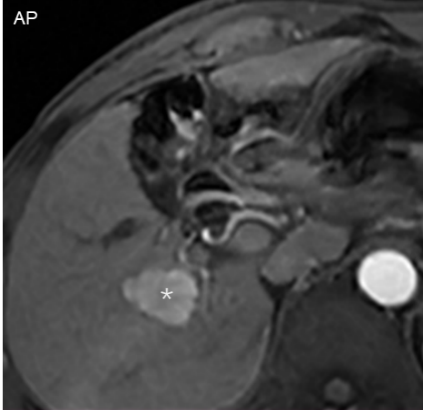 | |
|  |  | Present | | Absent | |
| **Fat in mass, more than adjacent liver** | Excess fat within a mass, in whole or in part, relative to adjacent liver [4] | 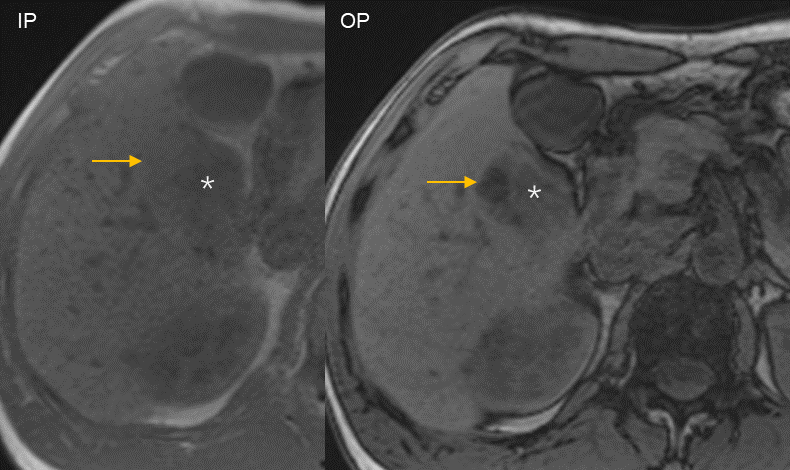 | | 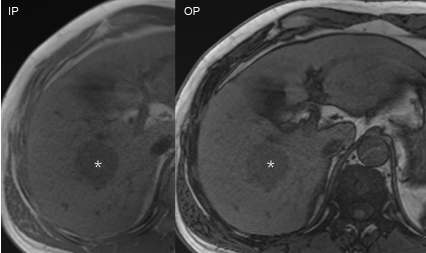 | |
|  |  | Present | | Absent | |
| **Blood products in mass** | Intralesional or perilesional hemorrhage in the absence of biopsy, trauma or intervention [4] | 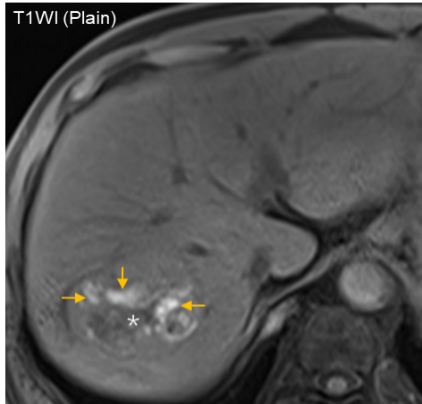 | | 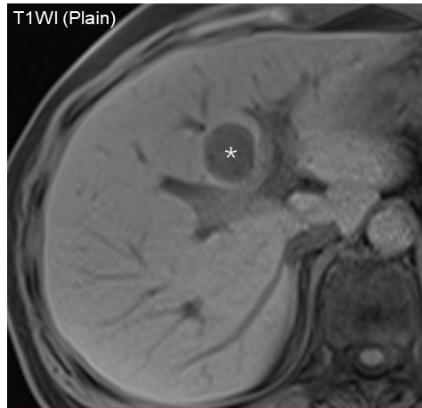 | |
|  |  | Present | | Absent | |
| **Parallels blood pool enhancement**^‡^ | Temporal pattern in which enhancement eventually reaches and then matches that of blood pool [4] | - | | | |
| **Undistorted vessels**^‡^ | Vessels traversing an observation without displacement, deformation, or other alteration [4] | - | | | |
| **Iron in mass, more than liver** | Excess iron in a mass relative to background liver [4] | 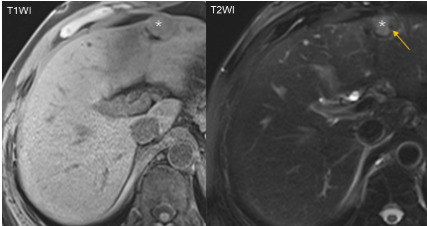 | | 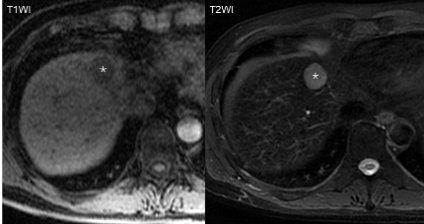 | |
|  |  | Present | | Absent | |
| **Marked T2 hyperintensity** | Signal intensity of the liver observation on T2 weighed-imaging markedly higher than liver and similar to bile ducts and other fluid-filled structures [4] | 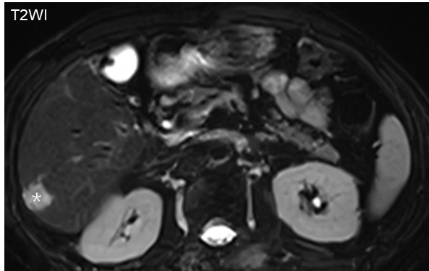 | | 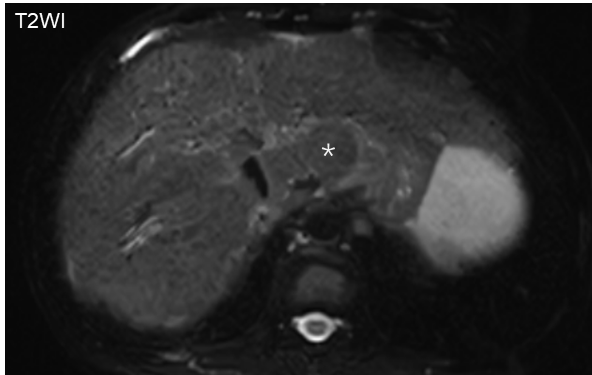 | |
|  |  | Present | | Absent | |
| **Hepatobiliary phase isointensity** | Signal intensity of the liver observation in the hepatobiliary phase nearly identical to liver [4] | 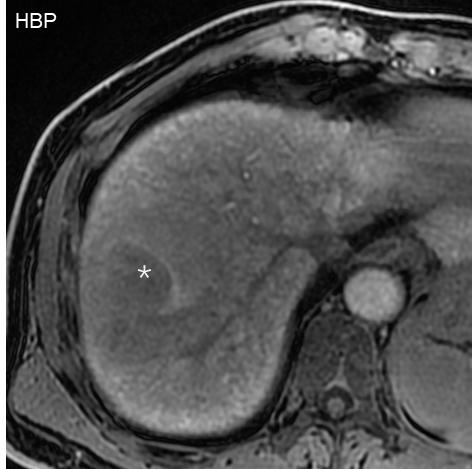 | | 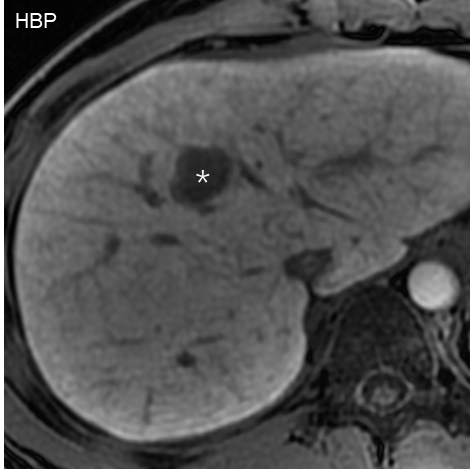 | |
|  |  | Present | | Absent | |
| **Tumor in vein** | Presence of unequivocal enhancing soft tissue in vein [4] | 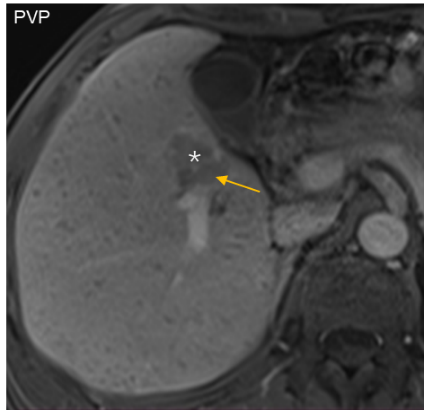 | | 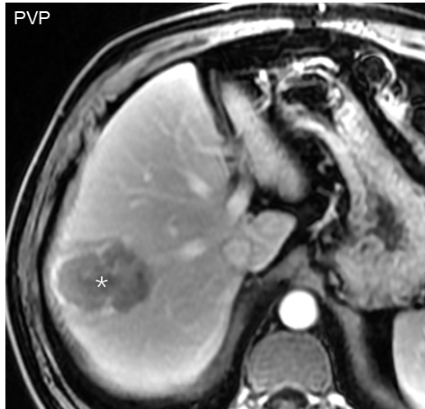 | |
|  |  | Present | | Absent | |
| **Rim arterial phase hyperenhancement** | Presence of arterial phase enhancement most pronounced in observation periphery [4] | 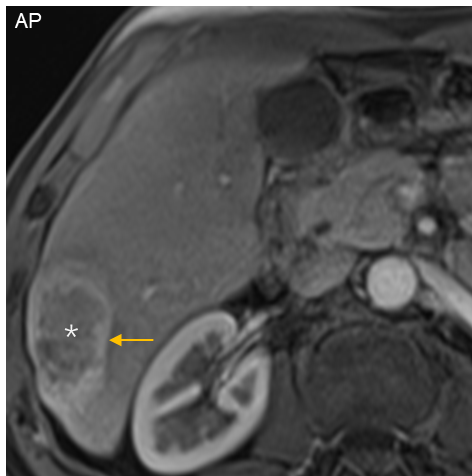 | | 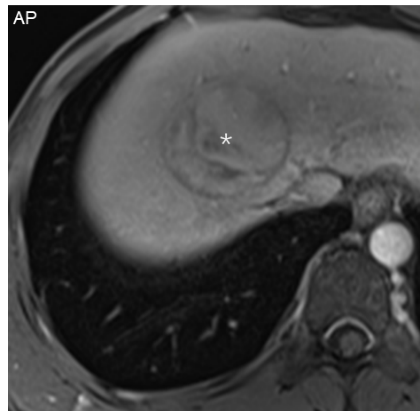 | |
|  |  | Present | | Absent | |
| **Peripheral "washout"** | Presence of apparent washout most pronounced in observation periphery [4] | 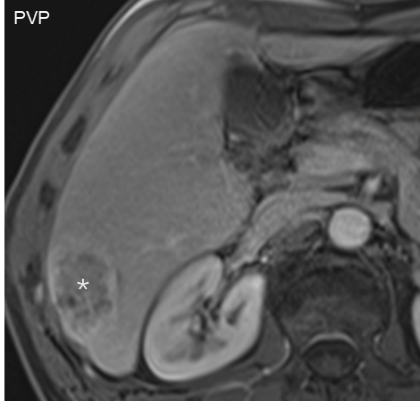 | | 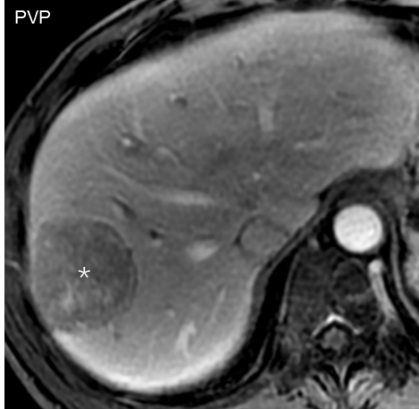 | |
|  |  | Present | | Absent | |
| **Delayed central enhancement** | Central area of progressive postarterial phase enhancement [4] | 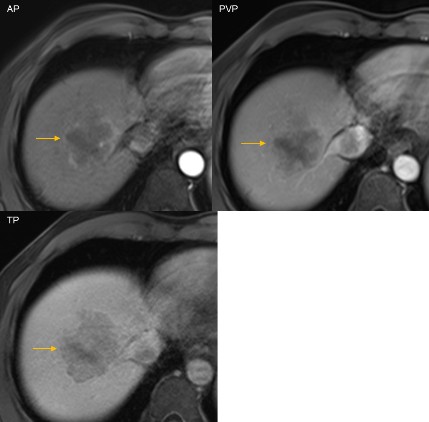 | | 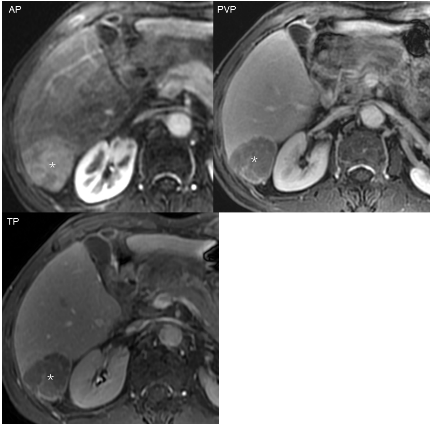 | |
|  |  | Present | | Absent | |
| **Targetoid restriction**^‡^ | Concentric pattern on diffusion-weighted imaging characterized by restricted diffusion in observation periphery with less restricted diffusion in observation center [4] | - | | | |
| **Targetoid TP or HBP appearance** | Concentric pattern in TP or HBP characterized by moderate-to-marked hypointensity in observation periphery with milder hypointensity in center [4] | 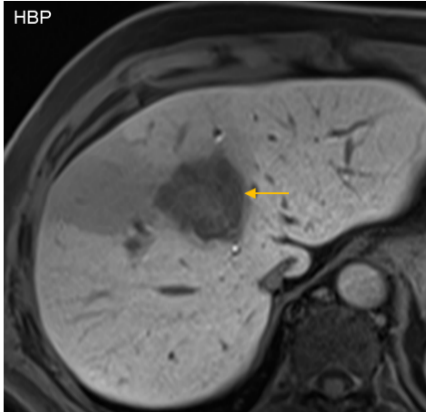 | | 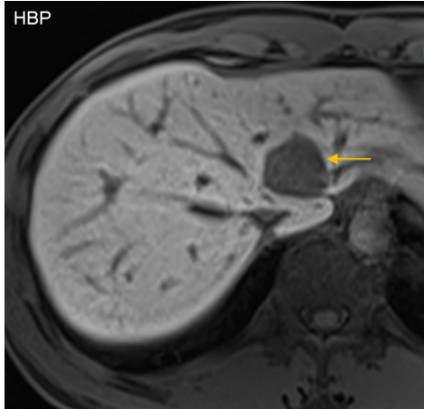 | |
|  |  | Present | | Absent | |
| **Infiltrative appearance** | Liver observation with non-circumscribed margin (indistinct transition) [4] | 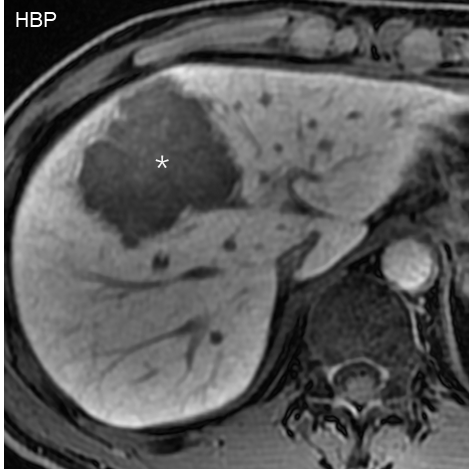 | | 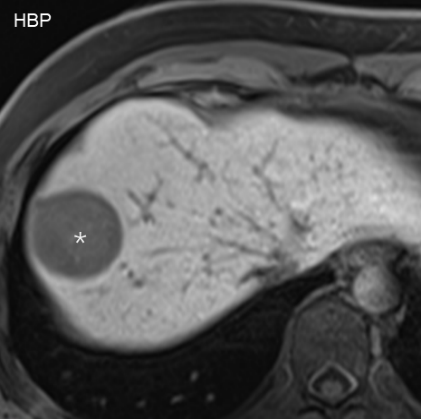 | |
|  |  | Present | | Absent | |
| **Marked diffusion restriction** | Increased signal intensity of the liver observation at diffusion-weighted imaging in relative to the spleen, not solely attributable to T2-weighted imaging shine-through effect [4] | 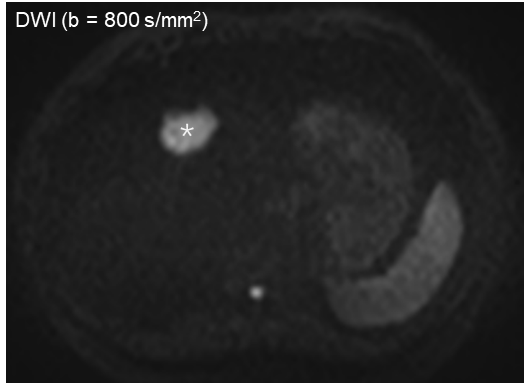 | | 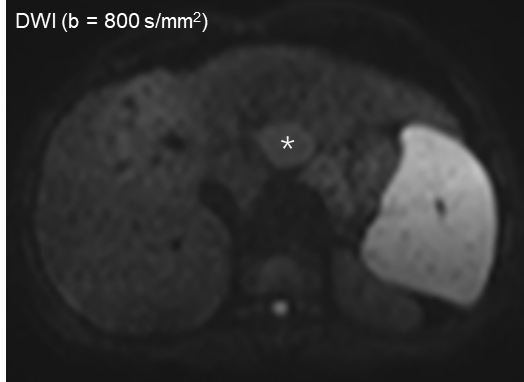 | |
|  |  | Present | | Absent | |
| **Necrosis or severe ischemia** | Presence of unequivocal intralesional necrosis or severe ischemia [4] | 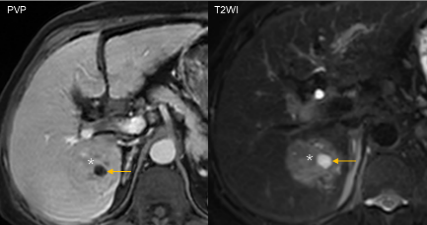 | | 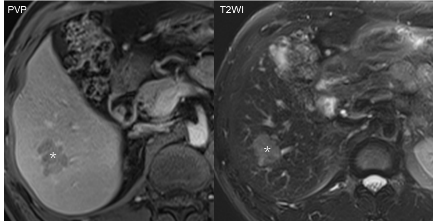 | |
|  |  | Present | | Absent | |
| **Liver surface retraction** | Presence of focal flattening or concavity of the normally convex hepatic contour [5] | 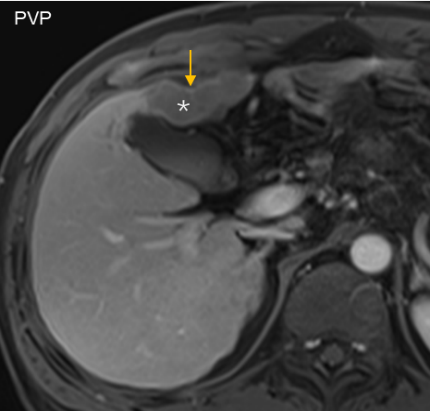 | | 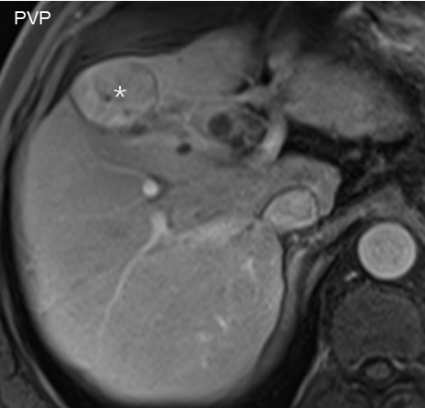 | |
|  |  | Present | | Absent | |
| **Adjacent biliary dilatation** | Presence of dilated bile ducts adjacent to the tumor border in any imaging plane | 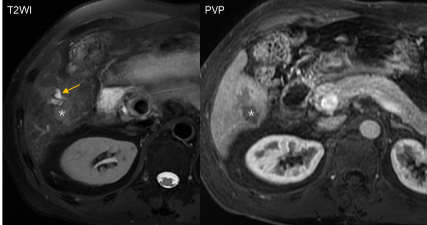 | | 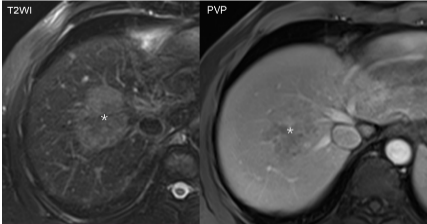 | |
|  |  | Present | | Absent | |
| **Radiologic cirrhosis**^§^ | An irregular, nodular or shrunken liver, as well as ascites or evidence of portosystemic collaterals in decompensated stage [6] | - | | | |
| **Bilobar involvement**^§^ | Bilobar involvement of definite HCC on gadoxetic acid-enhanced MR imaging | - | | | |
| **Internal artery** | Presence of discrete arterial enhancement within the tumor [7] | 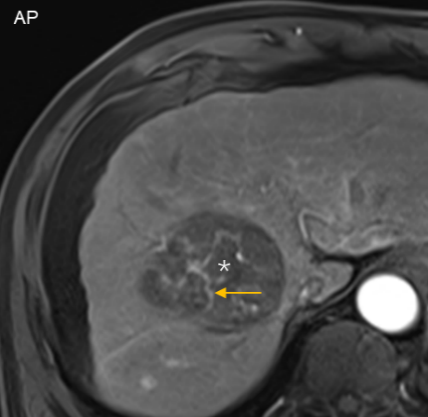 | | 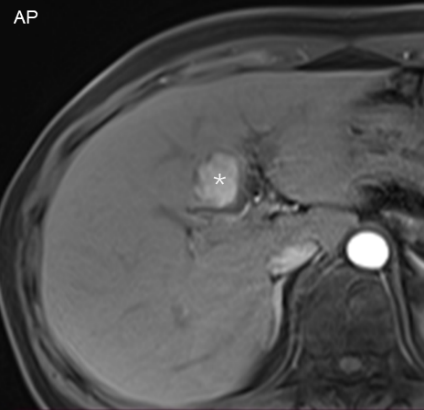 | |
|  |  | Present | | Absent | |
| **Non-smooth tumor margin** | Presence of non-nodular tumors with irregular contour that had budding portion at the periphery [8] | 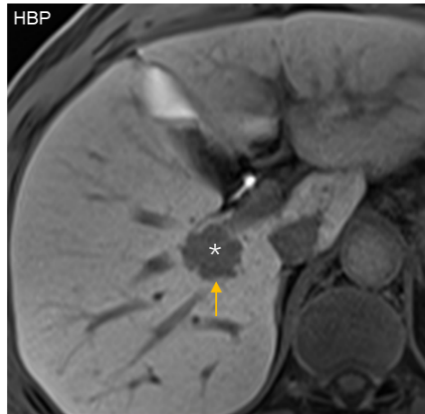 | | 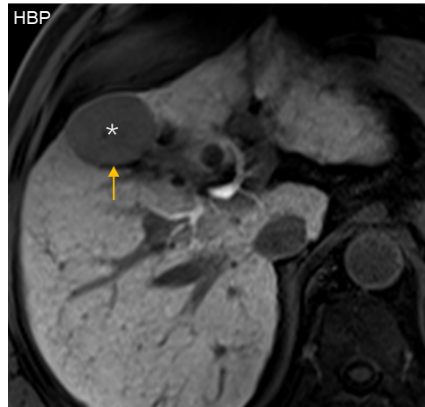 | |
|  |  | Present | | Absent | |
| **Peritumoral hypointensity on HBP** | Presence of wedge-shaped or flame-like hypointense area adjacent to the tumor border on hepatobiliary phase images [8] | 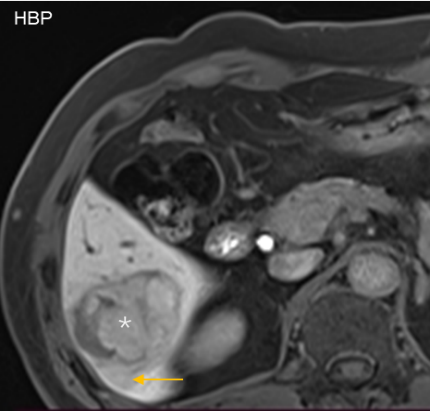 | | 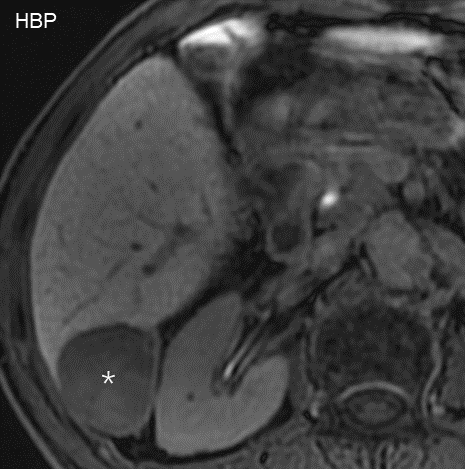 | |
|  |  | Present | | Absent | |
| **Tumor capsule** | -Complete: Presence of non-disrupted "capsule" in all imaging planes;  -Incomplete: Presence of disrupted "capsule" in all imaging planes [9] | 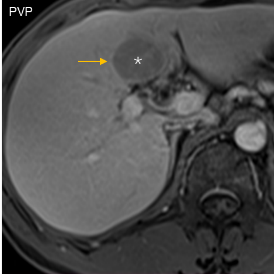 | 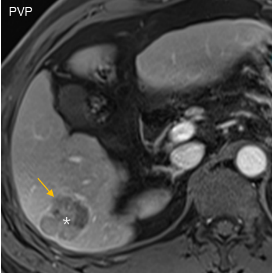 | | 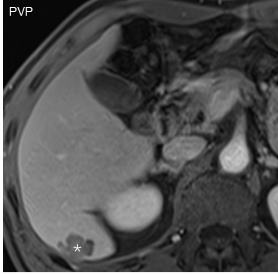 |
|  |  | Complete | Incomplete | | Absent |

EOB-MRI, gadoxetic acid–enhanced magnetic resonance imaging; HCC, hepatocellular carcinoma; PVP, portal venous phase; HBP, hepatobiliary phase; AP, arterial phase; TP, transitional phase; IP, in-phase; OP, opposed-phase; DWI, diffusion-weighted imaging; T2WI, T2-weighted imaging; T1WI, T1-weighted imaging; LI-RADS/LR, Liver Imaging Reporting and Data System.

^†^LI-RADS v2018 features correlated with growth or ultrasound visibility were not assessed due to lack of prior or concurrent ultrasound examinations.

^‡^Examples were not presented due to lack of corresponding imaging findings in the overall study cohort.

^§^Examples were omitted due to the limited space.

**References**

1. Marrero JA, Kulik LM, Sirlin CB et al (2018) Diagnosis, staging, and management of hepatocellular carcinoma: 2018 practice guidance by the American Association for the Study of Liver Diseases. Hepatology 68:723-750
2. European Association for the Study of the Liver (2018) EASL clinical practice guidelines: management of hepatocellular carcinoma. J Hepatol 69:182-236
3. Omata M, Cheng AL, Kokudo N et al (2017) Asia-Pacific clinical practice guidelines on the management of hepatocellular carcinoma: a 2017 update. Hepatol Int 11:317-370
4. CT/MRI Liver Imaging Reporting and Data System version 2018 (2018) Available via [https://www.acr.org/Clinical-Resources/Reporting-and-Data-Systems/LI-RADS/CT-MRI-LI-RADS-v2018. Accessed 25 Oct 2021](https://www.acr.org/Clinical-Resources/Reporting-and-Data-Systems/LI-RADS/CT-MRI-LI-RADS-v2018.%20Accessed%2025%20Oct%202021)
5. Blachar A, Federle MP, Sosna J (2009) Liver lesions with hepatic capsular retraction. Semin Ultrasound CT MR 30:426-435
6. Ji GW, Zhu FP, Xu Q et al (2020) Radiomic features at contrast-enhanced CT predict recurrence in early stage hepatocellular carcinoma: a multi-institutional study. Radiology 294:568-579
7. Renzulli M, Brocchi S, Cucchetti A et al (2016) Can current preoperative imaging be used to detect microvascular invasion of hepatocellular carcinoma? Radiology 279:432-442
8. Lee S, Kim SH, Lee JE, Sinn DH, Park CK (2017) Preoperative gadoxetic acid-enhanced MRI for predicting microvascular invasion in patients with single hepatocellular carcinoma. J Hepatol 67:526-534
9. Lei Z, Li J, Wu D et al (2016) Nomogram for preoperative estimation of microvascular invasion risk in hepatitis B virus-related hepatocellular carcinoma within the Milan criteria. JAMA Surg 151:356-363

**Table S3** Frequencies of EOB-MRI features

| Variable | Overall cohort  (n = 214) | Derivation set  (n = 150) | Test set  (n = 64) | *P* Value |
| --- | --- | --- | --- | --- |
| Tumor number |  |  |  | 0.181 |
| 1 | 145 (67.8) | 107 (71.3) | 38 (59.4) |  |
| 2 or 3 | 39 (18.2) | 23 (15.3) | 16 (25.0) |  |
| >3 | 30 (14.0) | 20 (13.3) | 10 (15.6) |  |
| LI-RADS v2018 feature^†^ | | | | |
| Major imaging feature | | | | |
| Size, cm | 4.1 (2.4-7.1) | 4.0 (2.4-7.2) | 4.4 (2.4-6.3) | 0.956 |
| Nonrim arterial phase hyperenhancement | 204 (95.3) | 142 (94.7) | 62 (96.9) | 0.729 |
| Nonperipheral "washout" | 198 (92.5) | 139 (92.7) | 59 (92.2) | 1.000 |
| Enhancing "capsule" | 143 (66.8) | 97 (64.7) | 46 (71.9) | 0.305 |
| Ancillary imaging feature | | | | |
| *Favoring malignancy in general, not HCC in particular* | | | | |
| Corona enhancement | 85 (39.7) | 54 (36.0) | 31 (48.4) | 0.089 |
| Fat sparing in solid mass | 12 (5.6) | 7 (4.7) | 5 (7.8) | 0.554 |
| Diffusion restriction | 214 (100.0) | 150 (100.0) | 64 (100.0) | … |
| Mild-moderate T2 hyperintensity | 211 (98.6) | 147 (98.0) | 64 (100.0) | 0.556 |
| Iron sparing in solid mass | 30 (14.0) | 19 (12.7) | 11 (17.2) | 0.383 |
| Transitional phase hypointensity | 211 (98.6) | 149 (99.3) | 62 (96.9) | 0.214 |
| Hepatobiliary phase hypointensity | 208 (97.2) | 147 (98.0) | 61 (95.3) | 0.523 |
| *Favoring HCC in particular* | | | | |
| Nonenhancing "capsule" | 40 (18.7) | 28 (18.7) | 12 (18.8) | 0.989 |
| Nodule-in-nodule | 75 (35.0) | 51 (34.0) | 24 (37.5) | 0.623 |
| Mosaic architecture | 97 (45.3) | 72 (48.0) | 25 (39.1) | 0.229 |
| Fat in mass, more than adjacent liver | 83 (38.8) | 62 (41.3) | 21 (32.8) | 0.241 |
| Blood products in mass | 81 (37.9) | 59 (39.3) | 22 (34.4) | 0.494 |
| *Favoring benignity* | | | | |
| Parallels blood pool enhancement | 0 (0.0) | 0 (0.0) | 0 (0.0) | … |
| Undistorted vessels | 0 (0.0) | 0 (0.0) | 0 (0.0) | … |
| Iron in mass, more than liver | 3 (1.4) | 2 (1.3) | 1 (1.6) | 1.000 |
| Marked T2 hyperintensity | 2 (0.9) | 2 (1.3) | 0 (0.0) | 1.000 |
| Hepatobiliary phase isointensity | 4 (1.9) | 3 (2.0) | 1 (1.6) | 1.000 |
| Tumor in vein | 43 (20.1) | 30 (20.0) | 13 (20.3) | 0.958 |
| LR-M criteria | | | | |
| *Targetoid appearance* | | | | |
| Rim arterial phase hyperenhancement | 8 (3.7) | 6 (4.0) | 2 (3.1) | 1.000 |
| Peripheral "washout" | 1 (0.5) | 1 (0.7) | 0 (0.0) | 1.000 |
| Delayed central enhancement | 2 (0.9) | 1 (0.7) | 1 (1.6) | 0.510 |
| Targetoid restriction | 0 (0.0) | 0 (0.0) | 0 (0.0) | … |
| Targetoid TP or HBP appearance | 2 (0.9) | 2 (1.3) | 0 (0.0) | 1.000 |
| *Nontargetoid LR-M feature* | | | | |
| Infiltrative appearance | 39 (18.2) | 27 (18.0) | 12 (18.8) | 0.896 |
| Marked diffusion restriction | 96 (44.9) | 66 (44.0) | 30 (46.9) | 0.699 |
| Necrosis or severe ischemia | 70 (32.7) | 49 (32.7) | 21 (32.8) | 0.983 |
| Liver surface retraction | 5 (2.3) | 3 (2.0) | 2 (3.1) | 0.996 |
| Adjacent biliary dilatation | 18 (8.4) | 11 (7.3) | 7 (10.9) | 0.384 |
| LIRADS category |  |  |  | 0.789 |
| LR-3 | 1 (0.5) | 1 (0.7) | 0 (0.0) |  |
| LR-4 | 11 (5.1) | 7 (4.7) | 4 (6.3) |  |
| LR-5 | 192 (89.7) | 134 (89.3) | 58 (90.6) |  |
| LR-M | 10 (4.7) | 8 (5.3) | 2 (3.1) |  |
| Other imaging feature | | | | |
| Radiologic cirrhosis | 93 (43.5) | 68 (45.3) | 25 (39.1) | 0.397 |
| Bilobar involvement | 24 (11.2) | 13 (8.7) | 11 (17.2) | 0.071 |
| Internal artery | 71 (33.2) | 51 (34.0) | 20 (31.3) | 0.696 |
| Non-smooth tumor margin | 133 (62.1) | 95 (63.3) | 38 (59.4) | 0.585 |
| Peritumoral hypointensity on HBP | 82 (38.3) | 59 (39.3) | 23 (35.9) | 0.640 |
| Tumor capsule |  |  |  | 0.336 |
| Absent | 31 (14.5) | 25 (16.7) | 6 (9.4) |  |
| Complete | 74 (34.6) | 49 (32.7) | 25 (39.1) |  |
| Incomplete | 109 (50.9) | 76 (50.7) | 33 (51.6) |  |

Data are expressed as n (%).

EOB-MRI, gadoxetic acid–enhanced magnetic resonance imaging; LI-RADS/LR, Liver Imaging Reporting and Data System; HCC, hepatocellular carcinoma; TP, transitional phase; HBP, hepatobiliary phase.

^†^LI-RADS v2018 features correlated with growth or ultrasound visibility were not assessed due to lack of prior and concurrent ultrasound examinations.

**Table S4** Univariable Cox regression analysis of predictors for recurrence on the derivation set

| Variable | Hazard ratio (95% CI) | *P* Value |
| --- | --- | --- |
| Clinical parameter |  |  |
| Age | 0.983 (0.961, 1.005) | 0.120 |
| Sex (male) | 1.761 (0.755, 4.108) | 0.191 |
| Etiology (non-HBV) | 1.759 (0.670, 4.421) | 0.230 |
| AST (>40 IU/L) | 2.591 (1.549, 4.335) | <0.001^*^ |
| ALT (>50 IU/L) | 2.310 (1.380, 3.866) | 0.001^*^ |
| TBIL (>19 µmol/L) | 0.862 (0.457, 1.628) | 0.648 |
| ALB (<40 g/L) | 1.044 (0.588, 1.856) | 0.882 |
| PLT (<100 x 10^9/L) | 0.541 (0.286, 1.021) | 0.058^*^ |
| PT (>13 s) | 0.893 (0.438, 1.818) | 0.754 |
| ALBI grade (3) | 1.055 (0.627, 1.775) | 0.839 |
| AFP (>400 ng/mL) | 2.667 (1.587, 4.481) | <0.001^*^ |
| Radiologic feature | | |
| Tumor number |  |  |
| 1 | … | … |
| 2 or 3 | 2.412 (1.243, 4.680) | 0.009^*^ |
| >3 | 3.542 (1.856, 6.763) | <0.001^*^ |
| Tumor diameter^†^ | 1.165 (1.094, 1.241) | <0.001^*^ |
| Enhancing "capsule" | 0.747 (0.438, 1.274) | 0.284 |
| Corona enhancement | 2.604 (1.547, 4.384) | <0.001^*^ |
| Iron sparing in solid mass | 1.474 (0.744, 2.919) | 0.266 |
| Nonenhancing "capsule" | 0.812 (0.411, 1.603) | 0.548 |
| Mosaic architecture^†^ | 2.018 (1.189, 3.424) | 0.009^*^ |
| Fat in mass, more than adjacent liver | 0.602 (0.351, 1.034) | 0.066^*^ |
| Blood products in mass | 2.011 (1.205, 3.356) | 0.007^*^ |
| Tumor in vein^‡^ | 3.483 (2.001, 6.064) | <0.001^*^ |
| Infiltrative appearance^‡^ | 6.678 (3.653, 12.211) | <0.001^*^ |
| Marked diffusion restriction | 1.765 (1.056, 2.950) | 0.030^*^ |
| Necrosis or severe ischemia | 1.062 (0.619, 1.822) | 0.826 |
| LIRADS category (LR-M) | 2.239 (0.889, 5.639) | 0.087^*^ |
| Radiologic cirrhosis | 0.799 (0.475, 1.344) | 0.398 |
| Internal artery | 2.452 (1.459, 4.119) | <0.001^*^ |
| Non-smooth tumor margin | 2.130 (1.198, 3.788) | 0.010^*^ |
| Peritumoral hypointensity on HBP | 2.589 (1.546, 4.334) | <0.001^*^ |
| Tumor capsule |  |  |
| Absent | … | … |
| Complete | 0.244 (0.109, 0.548) | <0.001^*^ |
| Incomplete | 0.609 (0.308, 1.202) | 0.153 |
| Pathologic characteristic | | |
| MVI | 2.830 (1.671, 4.791) | <0.001^*^ |
| Tumor differentiation (poor) | 1.764 (1.056, 2.945) | 0.030^*^ |

HBV, hepatitis B virus; AST, aspartate aminotransferase; ALT, alanine aminotransferase; TBIL, total bilirubin; ALB, albumin; PLT, platelet; PT, prothrombin time; ALBI, albumin-bilirubin; AFP, alpha-fetoprotein; LI-RADS/LR, Liver Imaging Reporting and Data System; HBP, hepatobiliary phase; MVI, microvascular invasion; CI, confidence interval.

^*^Variables with *P* < 0.1 in the univariable Cox regression analysis.

^†^Due to significant collinearity (*r_s_* = 0.767, *P* < 0.001), "mosaic architecture" was entered into the multivariable Cox regression model owing to the largest hazard ratio.

^‡^Due to significant collinearity (*r_s_* = 0.633, *P* < 0.001), "infiltrative appearance" was entered into the multivariable Cox regression model owing to the largest hazard ratio.

**Table S5** Prognostic performance of the preoperative score compared with the postoperative score and four clinical staging systems

| **Derivation set (n = 150)** | | | | | | | | | | | | |
| --- | --- | --- | --- | --- | --- | --- | --- | --- | --- | --- | --- | --- |
| Model | C-index  (95% CI) | *P* Value | 1-year tdAUC (95% CI) | *P* Value | 2-year tdAUC (95% CI) | *P* Value | 3-year tdAUC (95% CI) | *P* Value | 4-year tdAUC  (95% CI) | *P*  Value | 5-year tdAUC (95% CI) | *P*  Value |
| Score-  pre | 0.756  (0.695, 0.817) | ref | 0.796  (0.716, 0.875) | ref | 0.768  (0.678, 0.858) | ref | 0.803  (0.717, 0.888) | ref | 0.847  (0.759, 0.936) | ref | 0.914  (0.853, 0.975) | ref |
| Score-post | 0.770  (0.709, 0.831) | 0.863 | 0.808  (0.729, 0.887) | 0.497 | 0.793  (0.707, 0.879) | 0.324 | 0.835  (0.757, 0.913) | 0.182 | 0.871  (0.788, 0.954) | 0.492 | 0.899  (0.807, 0.992) | 0.718 |
| BCLC stage | 0.749  (0.669, 0.829) | 0.416 | 0.718  (0.634, 0.803) | 0.057 | 0.685  (0.592, 0.778) | 0.132 | 0.698  (0.605, 0.791) | 0.074 | 0.602  (0.480, 0.724) | 0.001^*^ | 0.525  (0.372, 0.679) | <0.001^*^ |
| HKLC stage | 0.739  (0.658, 0.820) | 0.321 | 0.717  (0.630, 0.803) | 0.110 | 0.643  (0.545, 0.740) | 0.025^*^ | 0.670  (0.572, 0.767) | 0.024^*^ | 0.625  (0.502, 0.748) | 0.004^*^ | 0.519  (0.336, 0.701) | <0.001^*^ |
| JIS score | 0.748  (0.671, 0.825) | 0.395 | 0.713  (0.629, 0.797) | 0.022^*^ | 0.691  (0.599, 0.783) | 0.120 | 0.724  (0.637, 0.810) | 0.128 | 0.637  (0.531, 0.744) | <0.001^*^ | 0.596  (0.466, 0.726) | <0.001^*^ |
| AJCC TNM stage | 0.743  (0.667, 0.819) | 0.339 | 0.721  (0.635, 0.807) | 0.049^*^ | 0.700  (0.609, 0.792) | 0.192 | 0.737  (0.652, 0.822) | 0.228 | 0.648  (0.539, 0.758) | 0.002^*^ | 0.573  (0.436, 0.711) | <0.001^*^ |
| **Test set (n = 64)** | | | | | | | | | | | | |
| Model | C-index  (95% CI) | *P* Value | 1-year tdAUC (95% CI) | *P* Value | 2-year tdAUC (95% CI) | *P* Value | 3-year tdAUC (95% CI) | *P* Value | 4-year tdAUC (95% CI) | *P*  Value | 5-year tdAUC (95% CI) | *P*  Value |
| Score-  pre | 0.741  (0.664, 0.818) | ref | 0.844  (0.745, 0.943) | ref | 0.726  (0.578, 0.874) | ref | 0.725  (0.568, 0.883) | ref | 0.678  (0.473, 0.884) | ref | 0.864  (0.696, 1.031) | ref |
| Score-post | 0.729  (0.646, 0.812) | 0.235 | 0.823  (0.717, 0.929) | 0.408 | 0.679  (0.521, 0.836) | 0.165 | 0.703  (0.539, 0.866) | 0.522 | 0.659  (0.444, 0.874) | 0.525 | 0.869  (0.704, 1.034) | 0.806 |
| BCLC stage | 0.720  (0.602, 0.839) | 0.327 | 0.751  (0.617, 0.885) | 0.069 | 0.669  (0.513, 0.826) | 0.372 | 0.718  (0.564, 0.872) | 0.896 | 0.659  (0.474, 0.844) | 0.763 | 0.837  (0.723, 0.950) | 0.768 |
| HKLC stage | 0.762  (0.651, 0.873) | 0.671 | 0.734  (0.600, 0.867) | 0.048^*^ | 0.692  (0.546, 0.839) | 0.576 | 0.700  (0.551, 0.849) | 0.650 | 0.615  (0.432, 0.799) | 0.304 | 0.724  (0.544, 0.903) | 0.159 |
| JIS score | 0.724  (0.605, 0.844) | 0.357 | 0.742  (0.607, 0.877) | 0.032^*^ | 0.681  (0.532, 0.831) | 0.434 | 0.718  (0.563, 0.873) | 0.898 | 0.657  (0.467, 0.848) | 0.720 | 0.854  (0.753, 0.955) | 0.912 |
| AJCC TNM stage | 0.712  (0.600, 0.825) | 0.257 | 0.736  (0.599, 0.873) | 0.036^*^ | 0.668  (0.514, 0.823) | 0.345 | 0.700  (0.544, 0.855) | 0.655 | 0.659  (0.484, 0.834) | 0.762 | 0.837  (0.723, 0.950) | 0.768 |

BCLC, Barcelona Clinic Liver Cancer; HKLC, Hong Kong Liver Cancer; JIS, Japan Integrated Staging; AJCC, American Joint Committee on Cancer; TNM, tumor-node-metastasis; tdAUC, area under the time-dependent receiver operating characteristic curve; CI, confidence interval.

^*^Statistically significant results.

**Table S6** Median RFS, 2- and 5-year RFS rates, and hazard ratio according to each risk group as defined by the preoperative score

| **Derivation set (n = 150)** | | | | | | |
| --- | --- | --- | --- | --- | --- | --- |
| Group | No. of patients | Median RFS, months (95% CI) | 2-year RFS rate, % (95% CI) | 5-year RFS rate, % (95% CI) | Hazard ratio (95% CI) | *P* Value |
| Low-risk group | 108 | 51.6  (35.7, NA) | 66.3  (56.8, 77.2) | 46.9  (35.3, 62.4) | 1 | <0.001^*^ |
| High-risk group | 42 | 6.0  (4.8, 9.4) | 18.1  (8.4, 39.0) | 6.0  (1.0, 35.5) | 4.253  (2.307, 7.838) |  |
| **Test set (n = 64)** | | | | | | |
| Group | No. of patients | Median RFS, months (95% CI) | 2-year RFS rate, % (95% CI) | 5-year RFS rate, % (95% CI) | Hazard ratio (95% CI) | *P* Value |
| Low-risk group | 40 | NA  (40.0, NA) | 74.8  (61.6, 90.9) | 55.4  (37.1, 82.7) | 1 | <0.001^*^ |
| High-risk group | 24 | 6.8  (3.0, 24.9) | 27.3  (13.8, 54.0) | 21.9  (9.7, 49.2) | 3.838  (1.731, 8.508) |  |

RFS, recurrence-free survival; NA, not applicable.

^*^Statistically significant results.

**Table S7** Aggressive pathologic features in the preoperative recurrence risk strata

| **Derivation set (n = 150)** | | | |
| --- | --- | --- | --- |
| Characteristic | Low-risk group | High-risk group | *P* Value |
| MVI |  |  | <0.001^*^ |
| Absent | 71 (65.7) | 12 (28.6) |  |
| Present | 37 (34.3) | 30 (71.4) |  |
| Tumor differentiation (poor) |  |  | 0.026^*^ |
| Absent | 75 (69.4) | 21 (50.0) |  |
| Present | 33 (30.6) | 21 (50.0) |  |
| **Test set (n = 64)** | | | |
| Characteristic | Low-risk group | High-risk group | *P* Value |
| MVI |  |  | <0.001^*^ |
| Absent | 29 (72.5) | 5 (20.8) |  |
| Present | 11 (27.5) | 19 (79.2) |  |
| Tumor differentiation (poor) |  |  | 0.015^*^ |
| Absent | 33 (82.5) | 13 (54.2) |  |
| Present | 7 (17.5) | 11 (45.8) |  |

Data are expressed as n (%).

MVI, microvascular invasion.

^*^Statistically significant results.


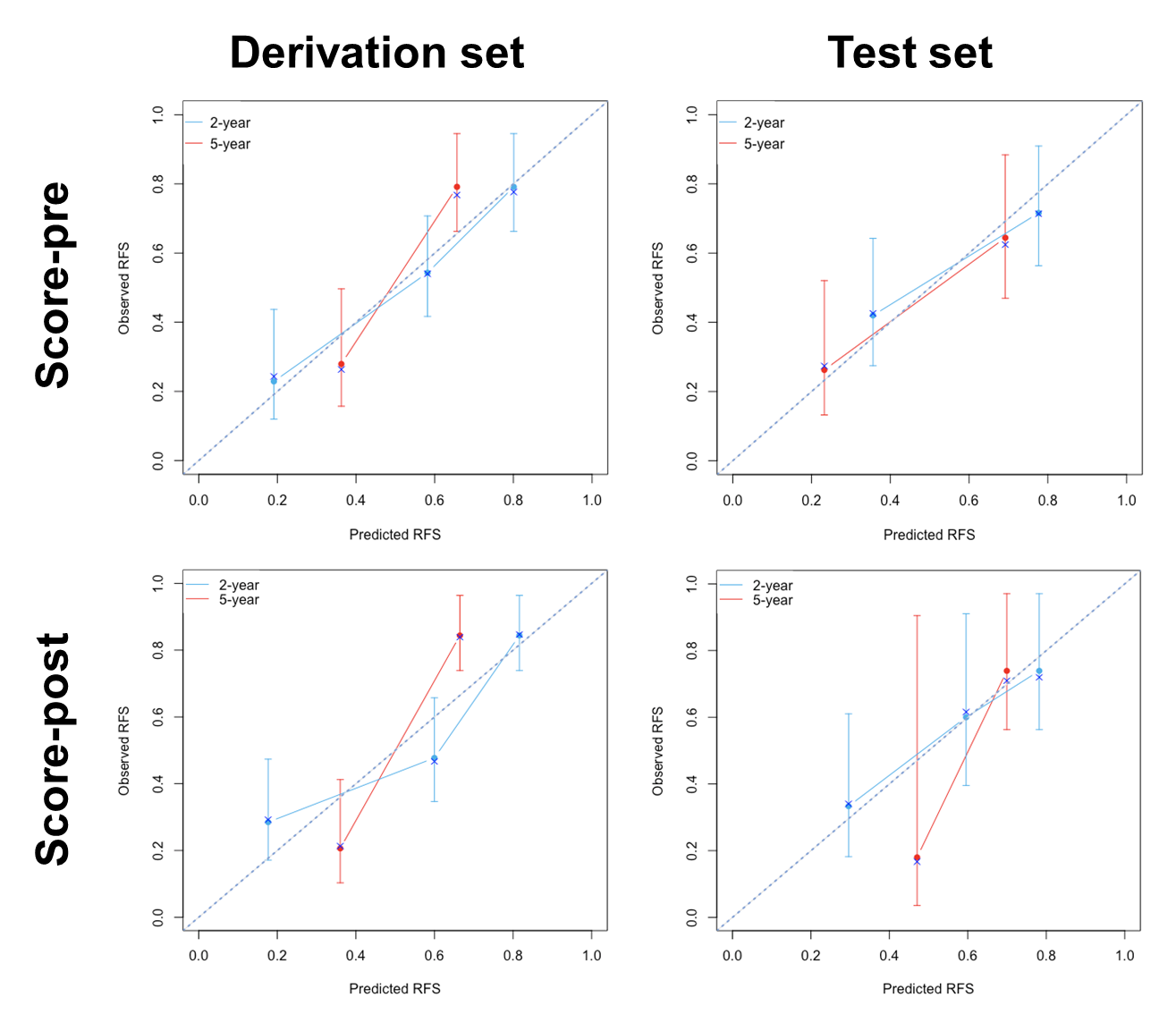


**Fig. S1** Calibration plots for 2- and 5-year RFS obtained by the preoperative and postoperative scores. RFS, recurrence-free survival.


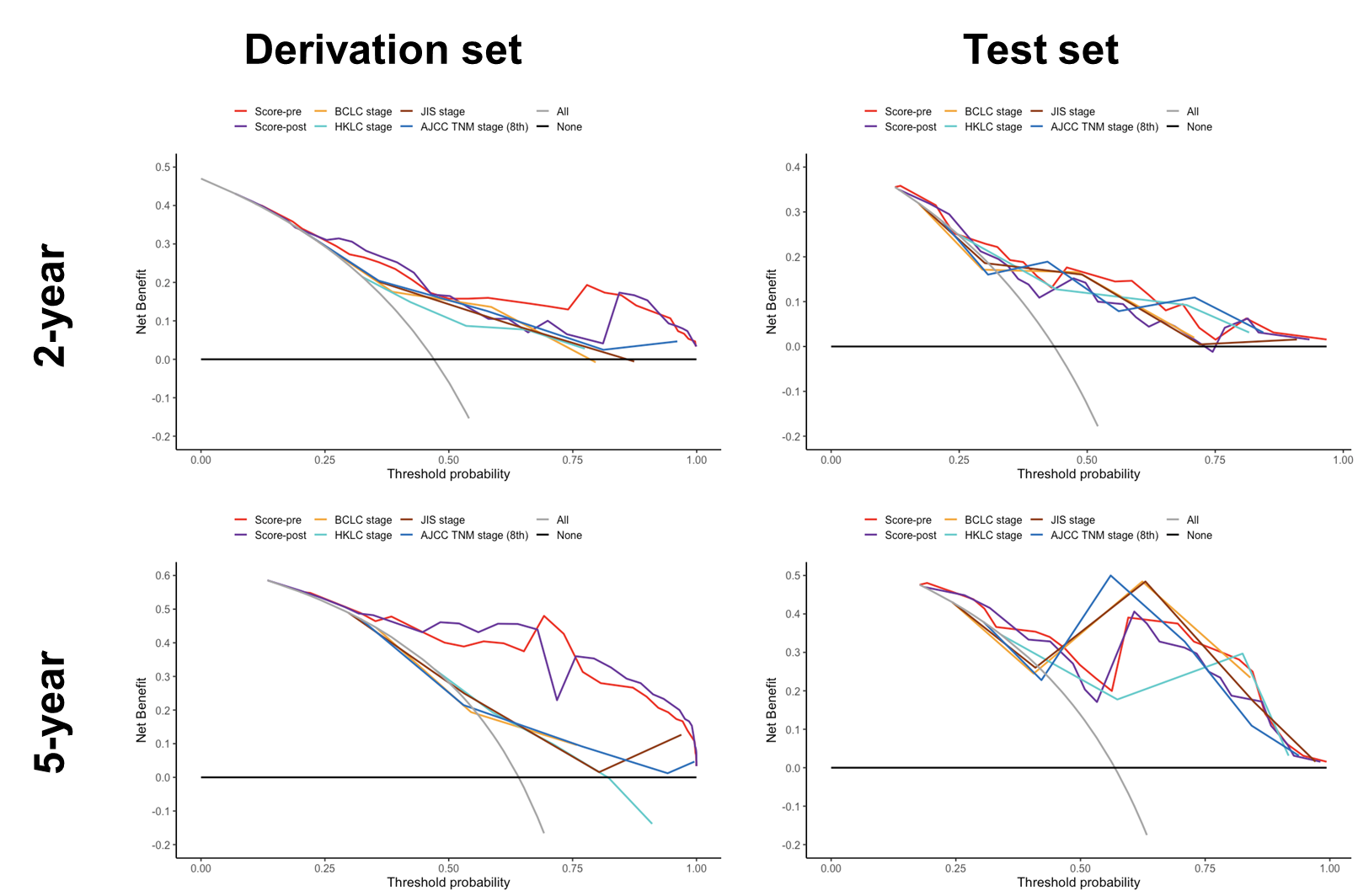


**Fig. S2** Decision curves for 2- and 5-year RFS obtained by the preoperative and postoperative scores and existing staging systems. BCLC, Barcelona Clinic Liver Cancer; HKLC, Hong Kong Liver Cancer; JIS, Japan Integrated Staging; AJCC, American Joint Committee on Cancer; TNM, tumor-node-metastasis; RFS, recurrence-free survival.
